# Sustained NF-κB activation allows mutant alveolar stem cells to co-opt a regeneration program for tumor initiation

**DOI:** 10.1016/j.stem.2025.01.011

**Published:** 2025-02-19

**Authors:** Frances J. England, Ignacio Bordeu, Minn-E Ng, JaeHak Bang, Bumsoo Kim, Jinwook Choi, Erik C. Cardoso, Bon-Kyoung Koo, Benjamin D. Simons, Joo-Hyeon Lee

**Affiliations:** 1 Cambridge Stem Cell Institute, Jeffrey Cheah Biomedical Centre, University of Cambridge, Cambridge, CB2 0AW, UK; 2 Gurdon Institute, University of Cambridge, Cambridge, CB2 1QN, UK; 3 Departamento de Física, Facultad de Ciencias Físicas y Matemáticas, Universidad de Chile, Santiago, Chile; 4 Center for Genome Engineering, Institute for Basic Science, Daejeon, 34126, Republic of Korea; 5 Department of Applied Mathematics and Theoretical Physics, Centre for Mathematical Science, University of Cambridge, Cambridge, CB3 0WA, UK; 6 Developmental Biology Program, Sloan Kettering Institute, Memorial Sloan Kettering Cancer Center, New York, NY 10065, USA

## Abstract

Disruptions to regulatory signals governing stem cell fate open the pathway to tumorigenesis. To determine how these programs become destabilized, we fate-map thousands of murine wildtype and *KrasG12D*-mutant alveolar type II (AT2) stem cells *in vivo* and find evidence for two independent AT2 subpopulations marked by distinct tumorigenic capacities. By combining clonal analyses with single-cell transcriptomics, we unveil striking parallels between lung regeneration and tumorigenesis that implicate *Il1r1* as a common activator of AT2 reprogramming. We show that tumor evolution proceeds through acquisition of lineage infidelity and reversible transitions between mutant states, which, in turn, modulate wildtype AT2 dynamics. Finally, we discover how sustained NF-κB activation sets tumorigenesis apart from regeneration, allowing mutant cells to subvert differentiation in favor of tumor growth.

Tissues deploy intricate reparative programs to restore homeostasis after injury. Feedback mechanisms play a crucial role in mediating this response by fine-tuning stem cell fate to ensure a balance between self-renewal and differentiation.^1–5^ However, tumors can hijack lineage-specific feedback loops to destabilize tissue equilibrium and aid disease progression.^6^ Determining how heterogenous cell populations utilize regulatory networks in health and disease is therefore critical for identifying therapeutic vulnerabilities and developing effective cancer preventative strategies.

In the distal gas-exchange regions of the adult lung, AT2 cells serve as the major stem cell population responsible for homeostatic turnover and tissue repair.^7,8^ These cells display functional heterogeneity during lung regeneration^8–11^, but due to the slow steady-state turnover of the alveolar epithelium, it remains unclear how distinct AT2 subpopulations function in unison to maintain the stem cell pool and contribute towards their alveolar type I (AT1) cell progeny. Dysregulation of homeostatic AT2 dynamics is closely linked to disease pathogenesis, with AT2 cells implicated as the key cell of origin for lung adenocarcinoma (LUAD)^12–14^, a leading cause of cancer-related mortality worldwide.^15,16^ Emerging studies have begun to unravel the transcriptomic and epigenomic landscape of LUAD^17–20^, with markers of cellular states associated with lung development and regeneration detected within LUAD tumors.^11,19,21,22^ These findings suggest exploitation of common regulatory pathways during tumor initiation, but the early cell fate transitions and molecular mechanisms differentiating oncogenic AT2 behavior from normal tissue responses remain unknown.

Here, we fate-map murine wildtype and *KrasG12D*-expressing mutant AT2 cells in parallel at clonal resolution. Together with comparative single-cell RNA sequencing (scRNA-seq) and organoid cultures, we demonstrate that mutant AT2 cells co-opt a regeneration program during tumorigenesis, which elicits a regenerative-like response in their wildtype neighbors. We show that lineage infidelity triggers stochastic reversible transitions that impart equivalent proliferative potential on heterogeneous mutant states to drive oncogenic expansion. We identify *Il1r1* as a common catalyst of AT2 reprogramming and reveal how sustained downstream NF-κB activation distinguishes oncogenesis from regeneration, highlighting a key target amenable to future therapeutic manipulation.

## RESULTS

### A two-population model encapsulates homeostatic AT2 cell behavior

**Figure 1.**
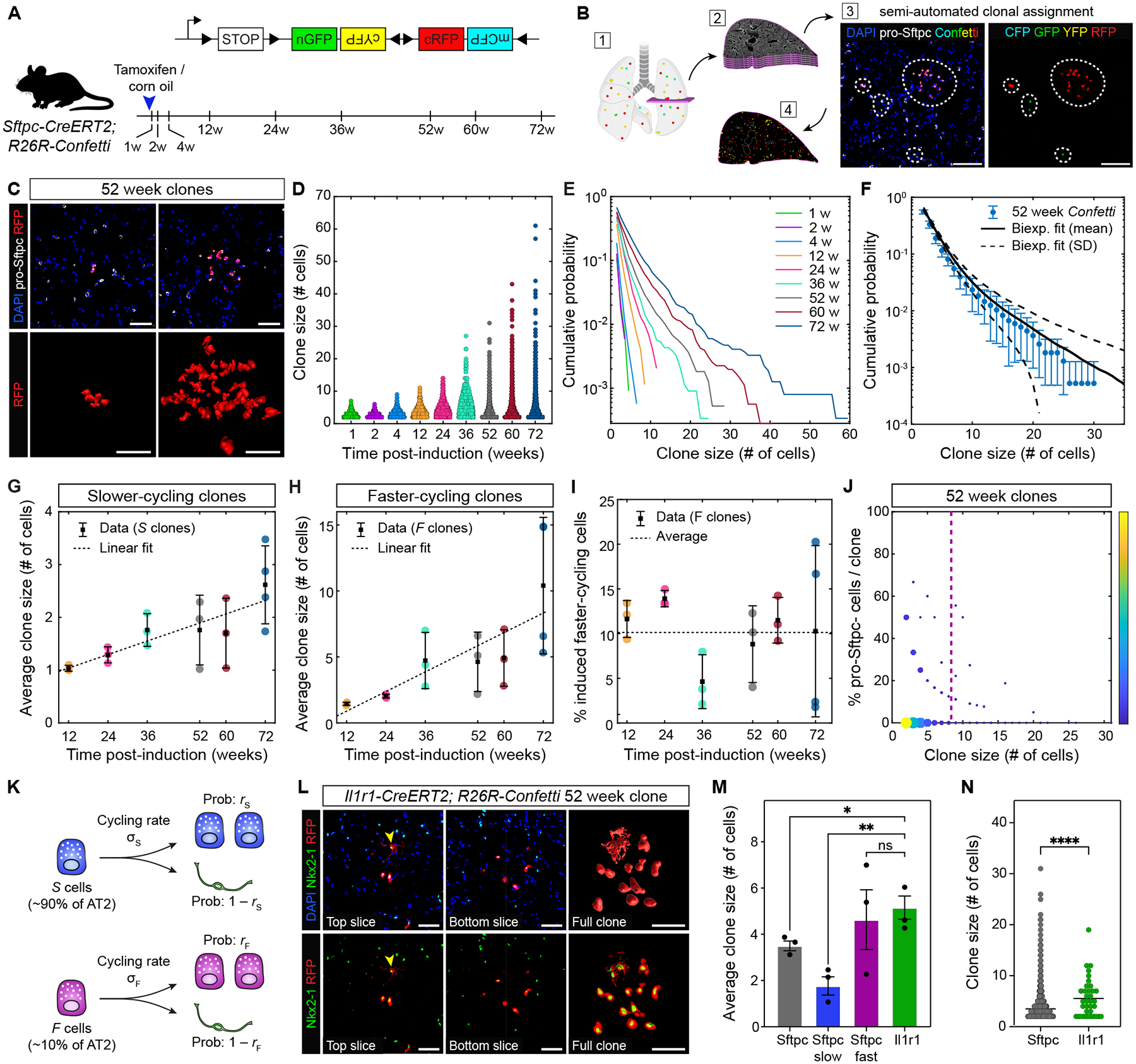
A two-population model encapsulates homeostatic AT2 cell dynamics. (**A**) Experimental design for lineage tracing homeostatic AT2-derived clones. (**B**) Schematic depicting the semi-automated fluorescence intensity-based pipeline used to assign clonal identity in each tissue section, producing 3D z-stack projections of RFP^+^ and YFP^+^ clones (step 4). (**C**) Representative confocal images of small and large 52-week homeostatic clones. Bottom panels represent 3D clonal reconstructions of top panels. DAPI (blue), pro-Sftpc (white), RFP (red). Scale bars, 50 μm. (**D**) Sizes of individual homeostatic clones. Each dot represents 1 clone. (**E**) Cumulative size distributions of homeostatic clones at each timepoint. Error bars omitted for clarity. (**F**) Cumulative size distribution of 52-week clones. Black line, biexponential fit; dashed line, SD of fit. (**G**), (**H**), Inferred average sizes of slower-cycling (G) and faster-cycling (H) clones. Each dot represents 1 mouse. Dashed line, linear fits. Data are represented as mean ± SD. (**I**) Inferred fraction of induced faster-cycling cells obtained from model fits (Methods S1). Dashed line, average value. Each dot represents 1 mouse. Data are represented as mean ± SD. (**J**) Percentage of pro-Sftpc^–^cells per 52-week homeostatic clone plotted against clone size. Color and size of dots are scaled according to clone density. Dashed line, boundary between slower- and faster-cycling clones. For (D)–(J), *n* ≥ 3 mice per timepoint (total numbers of clones and mice analyzed are given in Table S1). (**K**) Schematic depicting the minimal independent two-population AT2 model. *σ_S_* and *σ_F_* represent rates of cellular turnover, with *σ_F_* > *σ_S_* (Methods S1). (**L**) Representative confocal images of 52-week *Il1r1*^+^ AT2-derived clones. Left panels indicate top and bottom slices through the clonal z-stack. Rightmost panel shows the 3D clonal reconstruction. Yellow arrowhead, differentiating cell. DAPI (blue), Nkx2-1 (green), RFP (red). Scale bars, 50 μm. (**M**) Average sizes of 52-week *Sftpc-CreERT2;R26R-Confetti* clones in bulk (Sftpc) or split into slower (Sftpc slow) and faster (Sftpc fast) cycling fractions plotted alongside *Il1r1-CreERT2;R26R-Confetti* clones (Il1r1). Each dot represents 1 mouse. Data are represented as mean ± SEM. *n* = 3 mice. *p < 0.05; **p < 0.01, unpaired Student’s t-test. (**N**) Comparison of individual clone sizes from Sftpc and Il1r1 samples in (M). Each dot represents 1 clone. ****p < 0.0001, unpaired Student’s t-test. Images are representative of clones in *n* = 3 mice.

Distinct AT2 subpopulations have been identified^9–11^ but their kinetics and individual contributions to homeostatic AT2 and AT1 turnover have yet to be quantitatively defined. To establish a physiological baseline of AT2 stem cell behavior, we fate-mapped homeostatic AT2 cells at clonal resolution over 72 weeks in *Sftpc-CreERT2*;*R26R-Confetti* mice (Figure 1A). Initial lineage-labelled cells were of AT2 origin, with negligible quantities of background recombination in vehicle control littermates (Figures S1A–S1C). Although lung lobes gradually increased in size such that fewer pro-Sftpc^+^ cells were detected per field of view, their proportion relative to DAPI^+^ nuclei remained constant (Figures S1D–S1F), suggesting that increased lobe area reflected enlarged airspace rather than preferential AT2 expansion. Consistent with this deduction, the proportion of pro-Sftpc^+^ cells labelled with each fluorescent reporter remained stable over time (Figures S1G–S1I). These data verified tracing of a representative cohort of homeostatic AT2 cells, where any proliferation compensates for cell differentiation or loss.

To resolve AT2 cell dynamics, we developed a semi-automated intensity-based pipeline^23^ optimized for classifying clones in 3D from z-stack images acquired through 150 μm thick lung lobe cross-sections (Figure 1B). Although we observed a linear increase in average clone sizes over time, clone size distributions at each timepoint remained broad, with only a small subset of clones noticeably expanding (Figures 1C–1E and S1J). In addition, we noted a slight decline in clone density and detected rare AT2 cells expressing the apoptotic marker cleaved caspase-3 (Cl-Casp3), indicative of infrequent clone loss through apoptosis (Figures S1K–S1M). From 12 weeks post-induction onwards, the cumulative clone size distributions, representing the probability of finding a clone larger than a given size at a given timepoint, were found to be well-fitted by biexponential decays (Figures 1F, S1N, and S1O). These data suggested the co-existence of two independent AT2 subsets, each following a pattern of stochastic cell fate (Methods S1) and classified as either faster- or slower-cycling, with faster-cycling cells constituting a stable proportion of ~10% of AT2 cells (Figures 1G–1I).

To interrogate the differentiation potential of each AT2 subpopulation, we adapted our pipeline to extract information on cell fate, using loss of pro-Sftpc expression as a proxy for departure from AT2 identity. Although only 12.0±1.6% of lineage-labelled cells lost pro-Sftpc expression by the 72-week timepoint, pro-Sftpc^–^cells occurred in faster- and slower-cycling clones, signifying that differentiation was not limited to one AT2 subset (Figures 1J and S2A–S2F). Immunofluorescent analyses confirmed that pro-Sftpc^–^cells adopted a flattened morphology, becoming Ager^+^Cav-1^+^ AT1 cells (Figures S2G and S2H). Collectively, these results supported the two-population model comprised of faster-cycling (*F*) and slower-cycling (*S*) AT2 subpopulations (Methods S1), both of which have self-renewal and differentiation potential, but with the former turning over ~5 times faster than the latter (Figure 1K).

### Faster-cycling clones share characteristics with *Il1r1*^+^ AT2 cells

As the inferred proportion of faster-cycling cells resembled the fraction of *Axin2*^+^ and/or *Il1r1*^+^ AT2 cells previously shown to possess enhanced proliferative potential^9–11^, we investigated whether these populations overlapped. To this end, we traced homeostatic *Il1r1*^+^ AT2 cells at clonal resolution using *Il1r1-CreERT2*;*R26R-Confetti* mice (Figure S2I). Since *Il1r1* is expressed in a small fraction of non-epithelial alveolar cells^11^, only clones co-expressing the lung epithelial marker Nkx2-1 were included in our analyses, ensuring that all lineage-labelled clones were captured even once differentiation had occurred. We found that 52-week *Il1r1*^+^ AT2-derived clones were significantly larger and more homogenous in size than those in *Sftpc-CreERT2*;*R26R-Confetti* mice (Figures 1L–1N). Indeed, *Il1r1*^+^ AT2-derived clone sizes were statistically indistinguishable from those of faster-cycling clones (Figures 1M), suggestive of equivalent proliferative capacity in both populations. Nonetheless, *Il1r1*^+^ AT2-derived clones still showed evidence of differentiation, containing rare cells that morphologically resembled an AT1 phenotype (Figure 1L). These findings indicate that *Il1r1*^+^ AT2 cells exhibit similar homeostatic *in vivo* dynamics to faster-cycling cells, likely serving as the origin of at least part of this subpopulation.

To further characterize these dynamics, we used *Il1r1-CreERT2;R26R*-*ZsGreen* mice to label *Il1r1*^+^ AT2 cells in bulk before isolating the *Il1r1*^+^ and *Il1r1*^–^fractions for *in vitro* 3D organoid culture. Flow cytometry analyses verified that 6.12±3.71% of AT2 cells were *Il1r1*^+^ based on ZsGreen and MHCII expression^24^ (Figures S2J and S2K), congruent with the inferred fraction of faster-cycling cells. While ZsGreen^+^ and ZsGreen^–^cells both expressed AT2 markers, *Axin2* was specifically upregulated in the ZsGreen^+^ fraction (Figure S2L–N), suggesting that *Axin2*^+^ cells^9,10^ are enriched in this *Il1r1*^+^ subset. ZsGreen^+^ cells gave rise to considerably more organoids that were significantly larger than those derived from ZsGreen^–^and bulk AT2 cells (Figures S2O–S2Q). These findings corroborated our clonal-level data, denoting the existence of a faster-cycling subset likely derived from *Il1r1*^+^ AT2 cells, with shared expression of *Axin2*.

### Conservation of the two-population model following oncogenic perturbation

**Figure 2.**
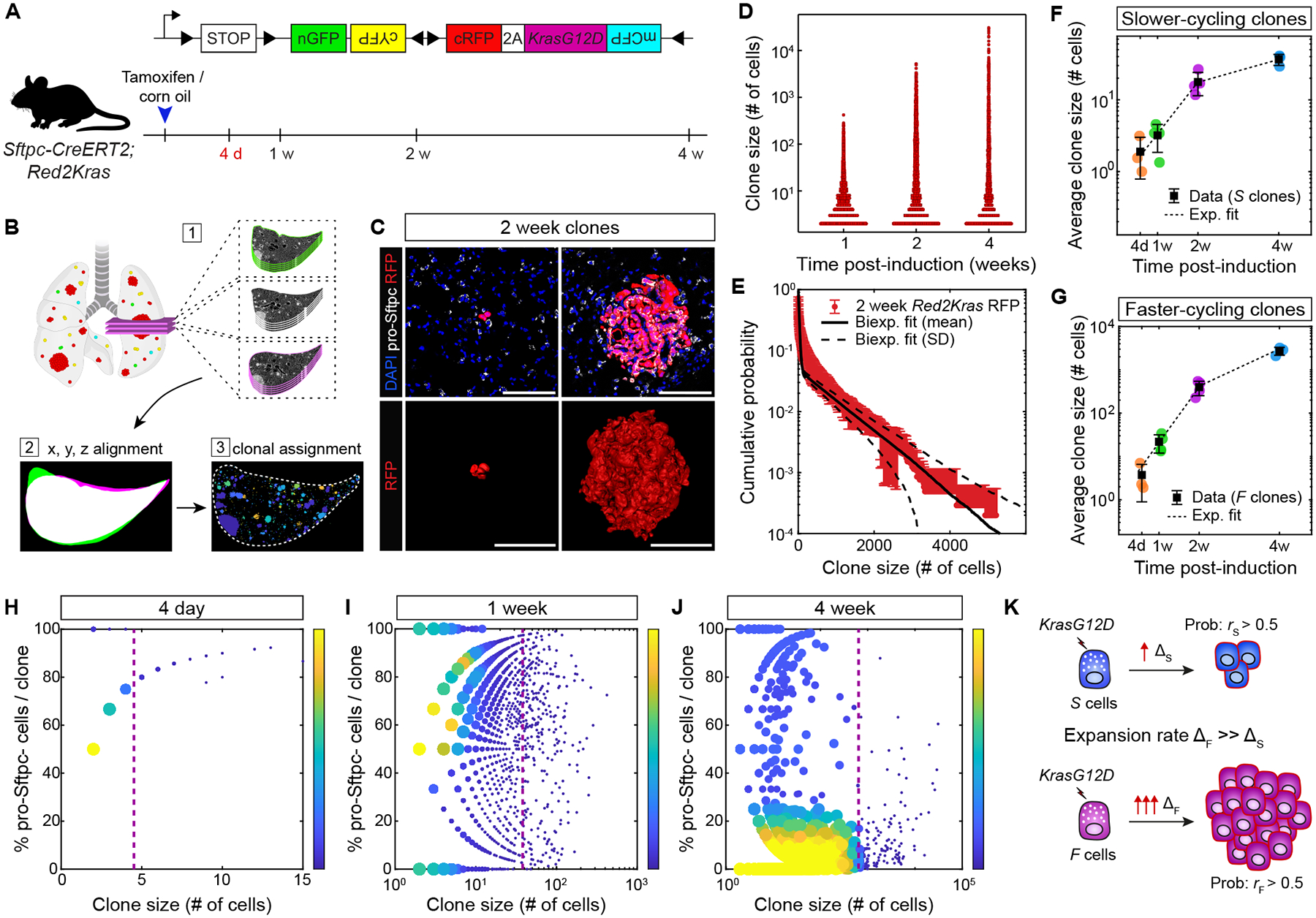
Two-population model dynamics are conserved in *KrasG12D*-driven tumor initiation. (**A**) Experimental design for tracing wildtype (GFP^+^, YFP^+^, and CFP^+^) and mutant (RFP^+^) AT2-derived clones using *Red2Kras* mice. (**B**) Schematic depicting the pipeline used to align and merge tiled z-stacks from consecutive *Red2Kras* tissue sections and perform clonal assignment, resulting in z-stack projections of mutant clones (step 3). (**C**) Confocal images of small and large 2-week mutant RFP^+^ clones. Bottom panels represent 3D clonal reconstructions of top panels. DAPI (blue), pro-Sftpc (white), RFP (red). Scale bars, 100 μm. Images representative of clones in *n* = 3 mice. (**D**) Sizes of individual mutant clones. Each dot represents 1 clone. (**E**) Cumulative size distribution of 2-week mutant clones. Black line, biexponential fit; dashed line, SD of fit. (**F**), (**G**), Inferred average sizes of slower-cycling (F) and faster-cycling (G) mutant clones. Each dot represents 1 mouse. Dashed line, exponential fits. (**H**), (**I**), (**J**), Percentage of pro-Sftpc^–^cells per mutant clone at 4 days (H), 1 week (I) and 4 weeks (**J**) post-induction. Color and size of dots are scaled according to clone density. Dashed line, boundary between slower- and faster-cycling clones. For (D)–(J), *n* ≥ 3 mice per timepoint (total numbers of clones and mice analyzed are given in Table S1). (**K**) Schematic depicting the impact of *KrasG12D* activation on the minimal two-population model in Figure 1K. Δ_S_ and Δ_F_ represent the expansion rate of each population, with Δ_F_≫Δ_S_ (Methods S1).

Next, we sought to understand how homeostatic dynamics are perturbed following acquisition of the LUAD driver mutation, *KrasG12D*, using the oncogene-associated multi-color reporter mouse model *Sftpc-CreERT2;Red2Kras*^25^ to simultaneously trace wildtype (GFP^+^, YFP^+^, and CFP^+^) and *KrasG12D*-expressing mutant (RFP^+^) AT2 cells at clonal resolution (Figure 2A). As expected, lineage-labelled cells expressed key AT2 markers, with mutant RasG12D and downstream phospho-ERK exclusively associated with the RFP reporter (Figures S3A, S3B, and Mendeley Data Figure 1), resulting in significant RFP^+^ cell expansion within 4 weeks (beyond which clone merger events became abundant) (Figures S3E–S3H).

Since mutant RFP^+^ clones often traversed multiple tissue sections, we adapted our clonal analysis pipeline to enable 3D reconstruction from z-stack images acquired through three consecutive lung lobe cross-sections (Figure 2B). Despite a significant increase in average mutant clone sizes, smaller clones remained abundant, while only a few substantially enlarged over the tracing period (Figures 2C–2E and S3H–S3J), corroborating previous reports.^8,12^ Importantly, the presence of small mutant clones could not be attributed to spontaneous recombination or a lack of RasG12D expression (Figure S3C and S3D), implying that AT2 founder cells have differing tumorigenic propensities. Consistent with this hypothesis, the cumulative clone size distributions were well-fitted by biexponential decays (Figures 2E, S3I and S3J), suggesting that the two-population model was retained after oncogenic perturbation. Moreover, while both faster- and slower-cycling mutant fractions exhibited exponential-like growth, surpassing their homeostatic growth rates, the expansion rate of faster-cycling clones was disproportionately higher, consistent with enhanced tumor initiation capacity (Figures 2F and 2G). By segregating mutant clones into their AT2-derived subsets, we estimated that some 6% were of faster-cycling origin, equivalent to the inferred proportion of faster-cycling cells comprising the *Red2Kras* wildtype YFP^+^ population (Figures S3K and S6D).

Analyses of clone compositions revealed a rapid loss of pro-Sftpc within most mutant clones at early timepoints (Figures 2H–2J, S3L, and Mendeley Data Figure 1). However, larger mutant clones at later timepoints tended to be enriched in pro-Sftpc^+^ cells (Figures 2J, S3L, and S3M), suggesting that early loss of AT2 identity may be followed by dynamic fluctuations in cell state. AT1 markers, such as Ager, were detected within some of these clones, but these Ager^+^ cells remained cuboidal and typically co-expressed AT2 markers, signifying incomplete differentiation (Figure S3N). We thus evaluated the levels of Cl-Casp3 to determine whether mutant cells might be lost via apoptosis instead. Interestingly, whilst apoptotic events were rare in large mutant clones, Cl-Casp3^+^ cells were readily detected within small mutant clones isolated from larger mutant regions, coinciding with a slight decrease in RFP^+^ clone density (Figures S3O–S3Q). Altogether, these data were consistent with a modified two-population model where all mutant AT2-derived clones undergo elevated expansion and reprogramming following *KrasG12D* activation, but where the faster-cycling population harbors enhanced tumorigenic capacity (Figure 2K).

### Oncogenic clones co-opt an alveolar regeneration program during tumor initiation

**Figure 3.**
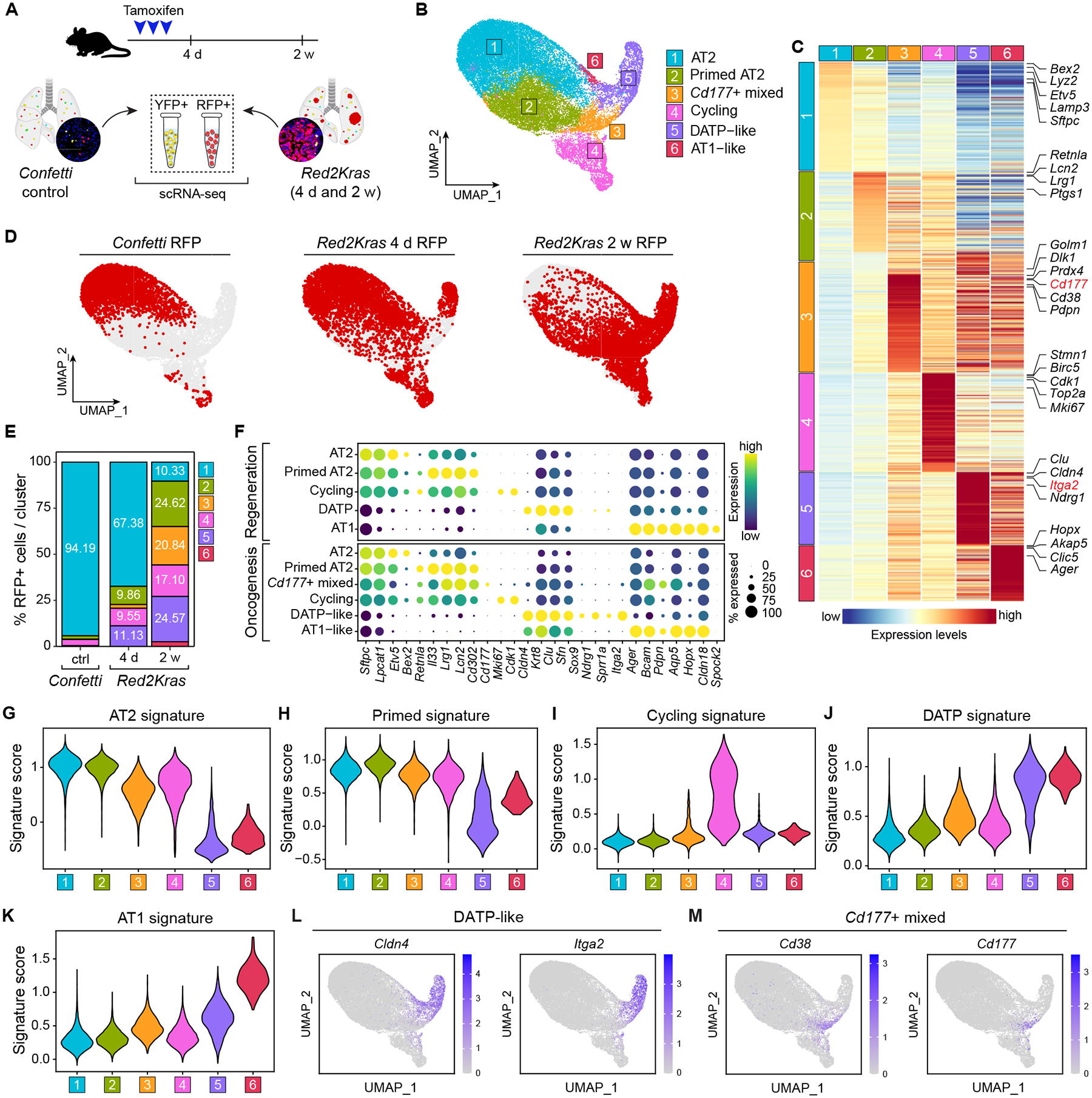
Mutant AT2 cells co-opt a regeneration program in tumorigenesis. (**A**) Experimental design for comparative scRNA-seq analyses of RFP^+^ and YFP^+^ *Confetti* and *Red2Kras* cells. (**B**) Uniform manifold approximation and projection (UMAP) visualization of all captured 29,563 cells from (A), clustered into six states. (**C**) Heatmap displaying expression of key marker genes distinguishing each state in (B). Genes highlighted in red represent cell surface markers used for cell state isolation. (**D**) Localization of RFP^+^ cells in each sample on the UMAP in (B). Red dots, cells in indicated sample; grey dots, all other cells. (**E**) Percentage of RFP^+^ cells occupying each state per sample in (D). Percentages greater than 5% are given within the corresponding bars in the plot. (**F**) Comparison of marker gene expression between states in alveolar regeneration^11^ (top) versus oncogenesis (bottom). Each dot is colored according to expression levels and sized according to the proportion of cells expressing that gene. (**G**), (**H**), (**I**), (**J**), (**K**), Violin plots showing the expression of AT2 (G), Primed (H), Cycling (I), DATP (J), and AT1 (K) signatures^11,26,27^ in each of the identified states in (B). (**L**), (**M**), UMAP plots showing expression of key DATP-like (L) and *Cd177*^+^ mixed (M) marker genes.

To delineate the fate transitions associated with mutant clone expansion, we conducted scRNA-seq on lineage-labelled cells isolated from *Sftpc-CreERT2;R26R-Confetti* (*Confetti*) and *Sftpc-CreERT2;Red2Kras* (*Red2Kras*) lungs 4 days and 2 weeks post-induction. Individual libraries were generated from mutant (*Red2Kras* RFP^+^) and wildtype (*Confetti* RFP^+^ and YFP^+^, and *Red2Kras* YFP^+^) epithelial cells and submitted for 10x Genomics sequencing, with the resultant 29,563 single-cell transcriptomic profiles spanning a total of six distinct clusters (Figures 3A–C and S4A). Extensive cellular reprogramming was evident across the mutant compartment from 4 days post-induction, with the proportion of RFP^+^ cells occupying the AT2 state dropping further to 10% by the 2-week timepoint (compared to 94% in *Confetti* controls) (Figures 3D and 3E). This loss of AT2 identity was accelerated relative to that in *Red2Kras* YFP^+^ cells (Figure S4F; see also Figure 6H), emphasizing a departure from wildtype fate dynamics.

Mutant reprogrammed states bore a striking resemblance to those arising during alveolar repair, with clusters exhibiting canonical markers of previously described Primed AT2 and DATP (Damage-Associated Transient Progenitor) populations^11^ (also termed “Activated AT2”^26^ and “PATS”^27^/“Krt8^+^ ADI”^26^), though with additional expression of *Itga2* in oncogenic DATP-like cells (Figures 3F–3M and S4B–S4K; see also Figures 7F–7H). In addition, we identified a *Cd177*^+^ mixed state enriched in the mutant population at later timepoints, which co-expressed AT2 and AT1 markers while also sharing features with other states (Figures 3C and 3F–3M). These *Cd177*^+^ mixed cells were exclusive to oncogenesis, with negligible *Cd177* expression detected in regeneration (Figure 3F; see also Figure 7G) and *Red2Kras* YFP^+^ controls (Figure S4F). Collectively, our data suggest that mutant AT2 cells initiate tumor formation by co-opting a regeneration program but later escape lineage fidelity, producing an aberrant *Cd177*^+^ mixed population.

### Reversible mutant state transitions lead to heterogeneous transcriptional signatures

**Figure 4.**
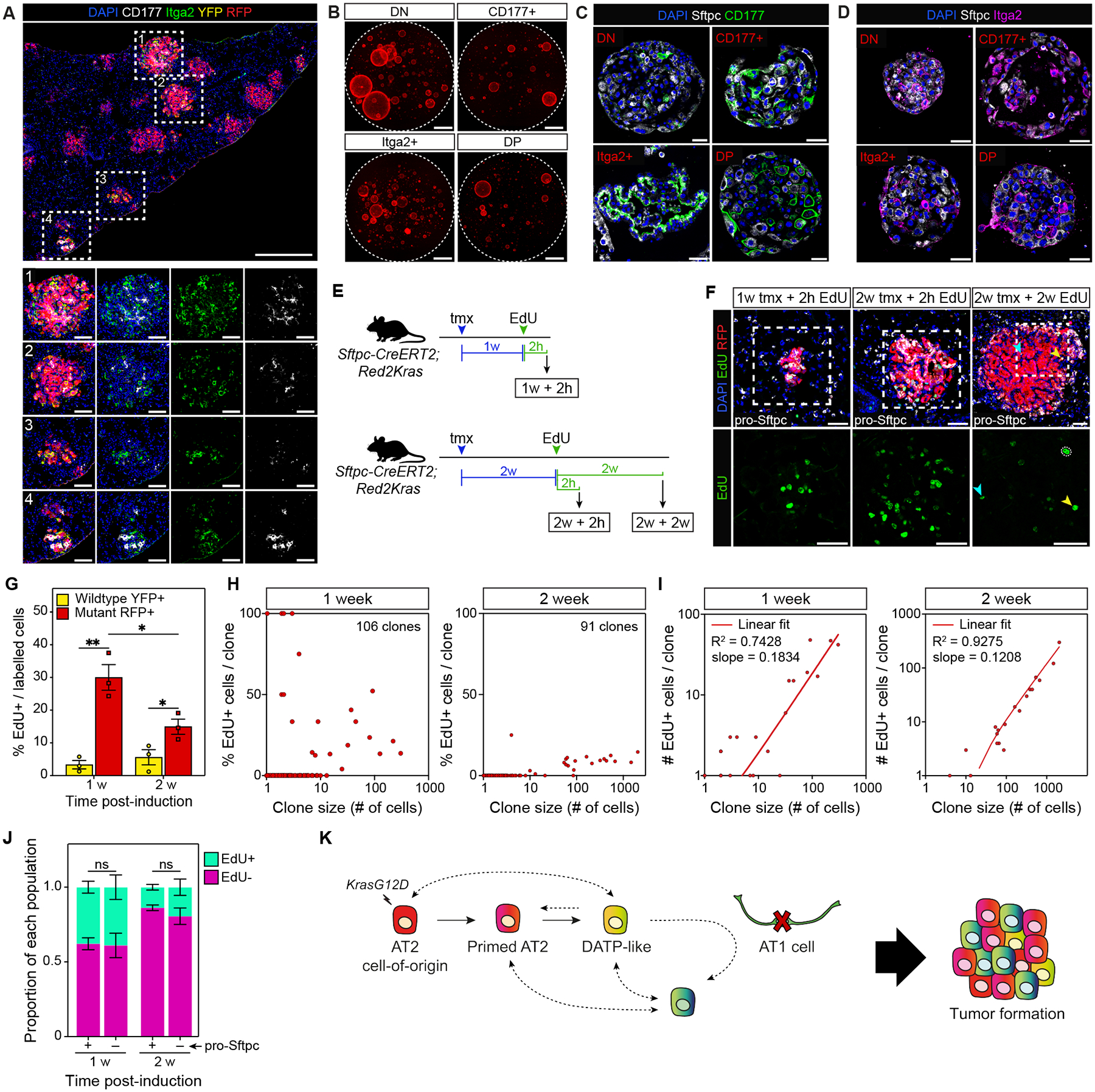
Reversible transitions between mutant states generate heterogeneous transcriptional signatures. (**A**) Tile scan showing Itga2 (green) and CD177 (white) expression in 4-week mutant clones (top panel) with magnifications of each (bottom panels). Tile scan representative of *n* = 3 mice 2- and 4-weeks post-induction. DAPI (blue), YFP (yellow), RFP (red). Scale bars, 200 μm (top), 50 μm (bottom). (**B**) Representative images of RFP^+^ organoids established from distinct mutant states. Dotted white circles, edge of well. Scale bars, 1000 μm. (**C**), (**D**) Representative confocal images of wholemount organoids in (B) showing expression of CD177 (green, (C)), Itga2 (magenta, (D)), and Sftpc (white, all panels). DAPI (blue). Scale bars, 50 μm. (**E**) Design of clonal EdU experiments performed 1- and 2-weeks post-induction, with data analyzed after 2 h and 2-week EdU chase periods. (**F**) Confocal images showing EdU expression (green) in mutant clones from all conditions in (E). Yellow arrowheads, pro-Sftpc^+^ EdU LRCs; cyan arrowheads, pro-Sftpc^–^EdU LRCs. Dashed white circle, EdU label-retaining post-mitotic doublets. DAPI (blue), RFP (red), pro-Sftpc (white). Scale bars, 50 μm. Images representative of *n* = 3 mice. (**G**) Percentage of EdU^+^ cells in *Red2Kras* YFP^+^ (yellow bars) and RFP^+^ (red bars) populations following a 2 h EdU chase. Each dot represents 1 mouse. Data are represented as mean ± SEM. *p < 0.05; **p < 0.01, unpaired Student’s t-tests. (**H**) Percentage of EdU^+^ cells per clone plotted against clone size on a linear-log scale. Each dot represents 1 clone with 106 (1-week) and 91 (2-week) clones quantified. (**I**) Number of EdU^+^ cells per mutant clone plotted against clone size on a log-log scale. Only proliferating clones (≥ 1 EdU^+^ cells) are shown. Each dot represents 1 clone. Red lines, best fit linear regression. Slopes and coefficients of determination (R^2^) are given in the panels. (**J**) Proportion of pro-Sftpc^+^ (+) and pro-Sftpc^–^(–) RFP^+^ cells per clone that were EdU^+^ (green) or EdU^–^(magenta). Data are represented as mean ± SEM. ns, not significant, paired Student’s t-test. For (G)–(J), *n* = 3 mice per timepoint. (**K**) Schematic depicting the proposed mechanism of oncogenic AT2 reprogramming through regenerative-like states. Failure to differentiate results in aberrant acquisition of a *Cd177*^+^ mixed phenotype and reversible transitions between individual states (dashed arrows).

To determine the reprogramming trajectory underpinning mutant clone evolution, we performed immunofluorescent analyses using CD177 and Itga2 as cell surface markers for the *Cd177*^+^ mixed and DATP-like states respectively. In accordance with our transcriptomic data, Itga2^+^ cells rapidly emerged within the mutant compartment, followed by CD177^+^RFP^+^ cells (Figure S5A). However, cells double positive for both markers at the protein level were also frequently observed, indicative of cells rapidly transitioning between the two transcriptional states (Figure S5A). To establish the sequence of cell state acquisition, we reasoned that if fate transitions progressed via a forward reprogramming trajectory, as in typical AT2 differentiation, larger clones would become enriched in cells occupying later states. However, we were unable to detect any correlation between clone size and clone composition within any mutant population either at the 2- or 4-week timepoints. Instead, clones of all sizes contained a wide variety of cellular states, irrespective of whether they were faster- or slower-cycling-derived (Figure 4A). Moreover, all states persisted within mutant clones long-term (Figure S5B), making a unidirectional reprogramming trajectory unlikely.

We therefore looked to functionally evaluate the plasticity of each state by isolating CD177^+^, Itga2^+^, double negative (DN), and double positive (DP) mutant RFP^+^ fractions for organoid culture (Figures S5C–S5G). RFP^+^ organoids were readily generated from all populations, with CD177^+^ and Itga2^+^ cells detected in all organoid cultures regardless of their state of origin (Figures 4B–D, S5H, and S5I), indicating that states harbored comparable levels of phenotypic plasticity. To ensure that these findings recapitulated *in vivo* cellular behavior, we orthotopically transplanted each mutant RFP^+^ state into immunodeficient mice and analyzed their fate via flow cytometry 2 weeks post-transplantation. As predicted, all 4 states emerged in roughly equal proportions within each engrafted RFP^+^ population (Figures S5J–S5M). Taken together, our results suggest a paradigm of oncogenic expansion whereby RFP^+^ cells frequently, yet stochastically, transition back and forth between mutant states, giving rise to heterogenous transcriptional signatures.

To interrogate the validity of this model, we investigated whether individual states imparted differing proliferative dynamics on mutant clones by combining clonal-level lineage tracing with EdU incorporation assays 1 and 2 weeks after tamoxifen induction (Figure 4E). Within 2 h of EdU administration, greater levels of proliferation were seen in mutant clones than in their wildtype YFP^+^ neighbors (Figures 4F and 4G). Although the proportion of EdU^+^ cells varied widely in 1-week mutant clones, 12.1±0.3% of cells in 2-week mutant clones were EdU^+^ irrespective of clone size (Figures 4H and 4I). Moreover, analogous quantities of EdU^+^ cells were detected in pro-Sftpc^+^ and pro-Sftpc^–^RFP^+^ fractions, with EdU^+^ label-retaining cells (LRCs) found in both populations 2 weeks later (Figures 4F, 4J, and S5N). However, some LRCs were captured undergoing post-mitotic separation (Figure 4F), indicating that they were not terminally differentiated. These data suggest that mutant cells initially transform along a forward reprogramming pathway but later gain capacity to undergo reversible transitions that impart equivalent proliferative potential on all mutant states (Figure 4K).

Given the behavioral similarities amongst mutant states, we sought to identify what functional roles they play in tumorigenesis through gene ontology enrichment analyses. Gene signatures significantly enriched in the DATP-like state were associated with focal adhesion, extracellular matrix organization, cellular migration, protein synthesis, and negative regulation of apoptosis (Figure S5O). Thus, this population may function to remodel their microenvironment, enable cell growth, and promote tumor survival and invasion. In contrast, cells in the *Cd177*^+^ mixed state were enriched in genes involved in an array of metabolic processes, response to oxidative stress, and protein localization to the telomere, indicating that they may be primed to sustain continual mutant cell division (Figure S5P). Considering that regenerative DATP/PATS/Krt8^+^ ADI cells (to which the DATP-like state bears some resemblance) were previously associated with a senescent phenotype^11,26,27^, our findings suggest that acquisition of the mutant-specific *Cd177*^+^ state may serve as an essential transitory step required to bypass senescence and retain proliferative potential. As such, rapid reversible state transitions may be required for persistent tumor growth and survival.

### Oncogenic clones directly modulate wildtype AT2 dynamics

**Figure 5.**
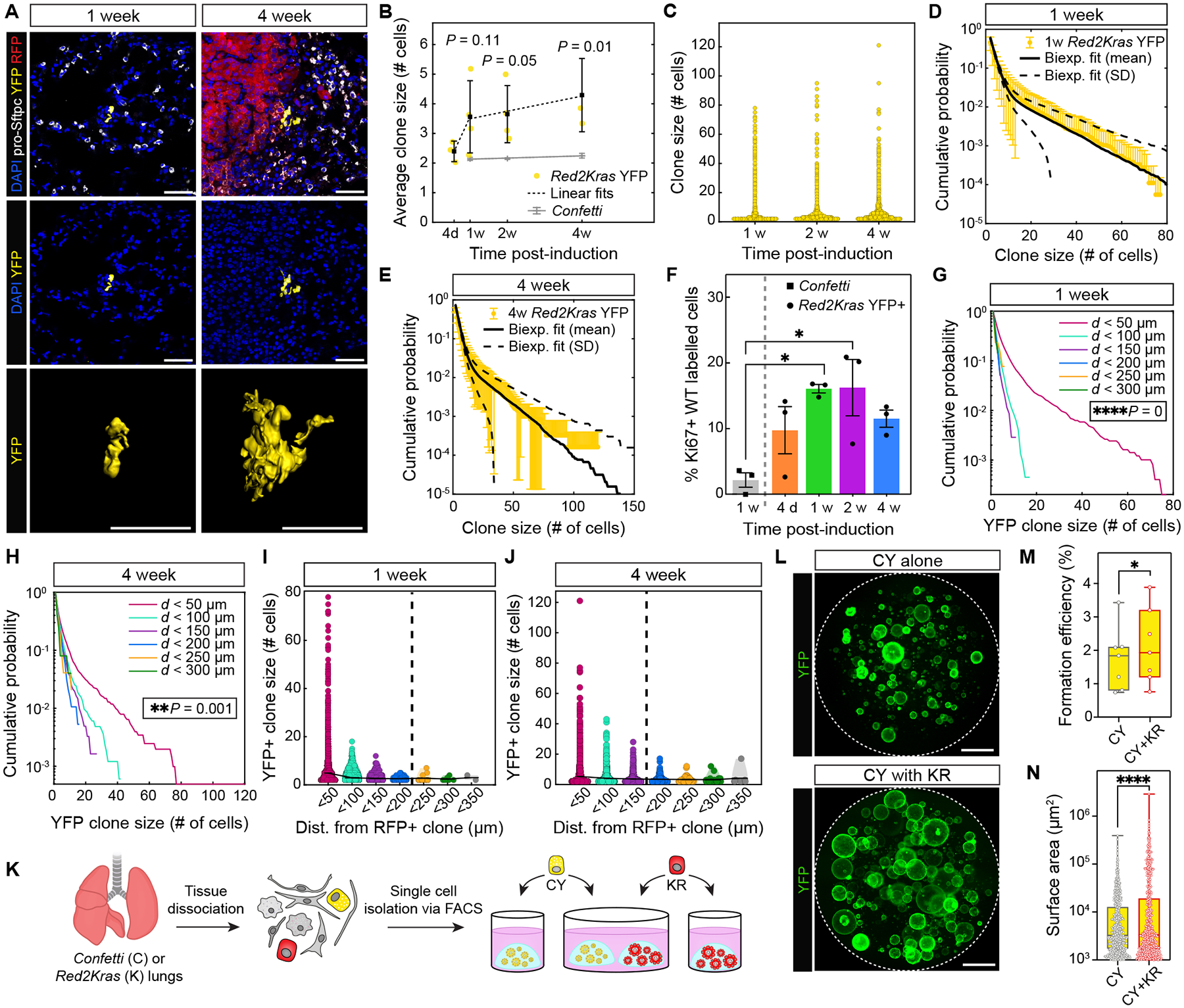
Oncogenic clones increase wildtype AT2 proliferation in a proximity-dependent manner. (**A**) Confocal images of 1-week (left) and 4-week (right) *Red2Kras* wildtype YFP^+^ clones. Bottom panels represent 3D clonal reconstructions of top panels. Images representative of expanding clones in *n* = 3 mice. DAPI (blue), pro-Sftpc (white), YFP (yellow), RFP (red). Scale bars, 50 μm. (**B**) Average *Red2Kras* wildtype clone sizes, with corresponding *Confetti* data in grey. Each dot represents 1 mouse. Dashed lines, linear fit. Data are represented as mean ± SD. *p* values given relative to *Confetti* data, unpaired Student’s t-tests. (**C**) Sizes of individual *Red2Kras* YFP^+^ clones at each timepoint. Each dot represents 1 clone. (**D**), (**E**) Cumulative size distributions of 1-week (D) and 4-week (E) *Red2Kras* wildtype clones. Black line, biexponential fit; dashed line, SD of fit. (**F**) Percentage of Ki67^+^ cells in wildtype (WT) *Confetti* (squares) or *Red2Kras* (circles) samples. Data are represented as mean ± SEM. Each symbol represents 1 mouse. *n* = 3 mice per timepoint. *p < 0.05 relative to 1-week *Confetti* data, one-way ANOVA with Dunnett’s multiple comparisons test. (**G**), (**H**) Cumulative size distributions of 1-week (G) and 4-week (H) YFP^+^ clones located at different radii, *d*, from the nearest mutant clone. Each line represents the average distribution of YFP^+^ clones at the given distance. *p* value is reported for the *d*<50 μm distribution relative to all others, Kolmogorov-Smirnov test. (**I**), (**J**) Sizes of individual 1-week (I) and 4-week (J) YFP^+^ clones at different radii in (G) and (H). Each dot represents 1 clone. Dashed line, point at which clone sizes become statistically indistinguishable from those in *Confetti* samples, Kolmogorov-Smirnov tests. For (D), (E), and (G)–(J), *n* ≥ 3 mice per timepoint (total numbers of clones and mice analyzed are given in Table S1). (**K**) Experimental protocol for establishing mutant:wildtype organoid cultures using freshly isolated *Confetti* and *Red2Kras* YFP^+^ and RFP^+^ cells. (**L**) Representative images of YFP^+^ AT2-derived organoids established from *Confetti* lungs and cultured alone (CY) or in the same well as mutant RFP^+^ organoids (CY+KR). Dotted white circles, edge of Matrigel dome. Scale bars, 1000 μm. (**M**) Formation efficiency of organoids in (L). Each dot represents 1 independent experiment. (**N**) 2D surface area of organoids in (L). Each dot represents 1 organoid, with 150 organoids quantified per experiment. For (M) and (N), *n* = 7 independent experiments. Centre line, median; box, interquartile range; whiskers, range. *p < 0.05; ***p < 0.0001 relative to YFP^+^ organoids cultured alone, paired Student’s t-tests.

Next, we leveraged the *Red2Kras* system for comparative analyses to assess whether these rapid oncogenic transformation events influenced the dynamics of tissue-sharing wildtype AT2 cells, using the *Confetti* dataset as an external homeostatic control. We confirmed that lineage-labelled *Red2Kras* wildtype AT2 cells exhibited similar dynamics to their unlabeled counterparts (Figures S6A and S6B). Then, we employed our clonal analysis pipeline to identify oncogene-induced changes in wildtype behavior, focusing on the abundant YFP^+^ fraction. We found that *Red2Kras* YFP^+^ clones were significantly larger on average than corresponding homeostatic clones, with those at the 4-week timepoint akin to 72-week *Confetti* clones (Figures 5A–5C and S1J). Nonetheless, heterogeneity in YFP^+^ clone sizes persisted across all timepoints (Figures 5D, 5E, and S6C), with cellular turnover rates proportionally elevated in slower- and faster-cycling fractions (Figures S6E and S6F). Consistent with these findings, we observed a significant increase in proliferating *Red2Kras* Ki67^+^YFP^+^ cells 1- and 2-weeks post-induction (Figure 5F). This wildtype expansion scaled with proximity to mutant clones, such that YFP^+^ clones situated within a 50 μm radius were significantly larger than those farther away, with clone sizes gradually approaching those of *Confetti* controls as the radius increased (Figures 5G–5J, S6G, and S6H). Moreover, *Confetti* YFP^+^ cells cultured in the same well, but in separate Matrigel domes, as mutant RFP^+^ cells formed significantly larger organoids with greater efficiency than those cultured alone (Figures 5K–5N). These results demonstrate that mutant cells directly modulate the stem cell activity of their wildtype neighbors in a distance-dependent manner.

### Oncogenic clones activate a regenerative program in wildtype AT2 neighbors

**Figure 6.**
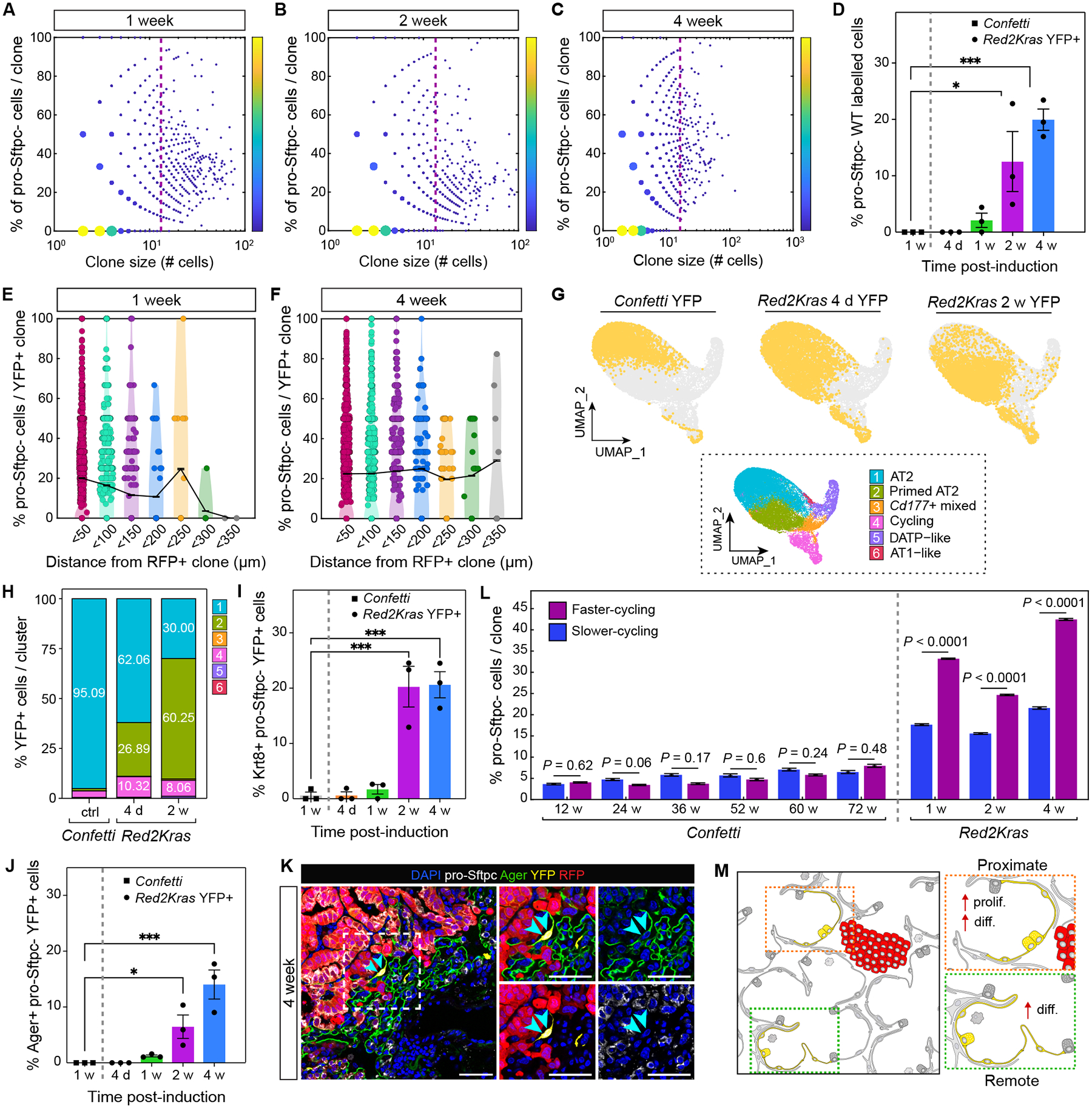
Oncogenic clones trigger a regenerative response in tissue-sharing wildtype AT2 cells. (**A**), (**B**), (**C**) Percentage of pro-Sftpc^–^cells in 1-week (A), 2-week (B), and 4-week (C) *Red2Kras* YFP^+^ clones plotted against clone size. Color and size of dots are scaled according to clone density. Dashed line, boundary between slower- and faster-cycling clones. (**D**) Percentage of pro-Sftpc^–^cells in wildtype (WT) lineage-labelled *Confetti* (squares) and *Red2Kras* (circles) populations. Data are represented as mean ± SEM. Each symbol represents 1 mouse. *n* = 3 mice per timepoint. *p < 0.05; ***p < 0.001 relative to 1-week *Confetti* data, one-way ANOVA with Dunnett’s multiple comparisons test. (**E**), (**F**) Percentage of pro-Sftpc^–^cells in 1-week (E) and 4-week (F) YFP^+^ clones located within different radii of mutant clones. Each dot represents 1 clone. (**G**) Localization of YFP^+^ transcriptional profiles (Figure 3A) on the UMAP. Yellow dots, cells in indicated sample; grey dots, all other cells. Inset, reference UMAP from Figure 3B showing cluster identity. (**H**) Percentage of YFP^+^ cells occupying each state per sample. Percentages greater than 5% are given within the corresponding bars in the plot. (**I**), (**J**) Percentage of pro-Sftpc^–^cells that were Krt8^+^ (I) and Ager^+^ (J) in *Confetti* (squares) and *Red2Kras* (circles) YFP^+^ populations. Data are represented as mean ± SEM. Each symbol represents 1 mouse. *n* = 3 mice per timepoint. *p < 0.05, ***p < 0.001 relative to 1-week *Confetti* data, one-way ANOVA with Dunnett’s multiple comparisons test. (**K**) Confocal image of flattened, elongated YFP^+^ cells expressing Ager (green), but not pro-Sftpc (white) (cyan arrowheads) in 4-week *Red2Kras* lungs. Right panels are magnifications of left panel. Image representative of *n* = 3 mice. DAPI (blue), YFP (yellow), RFP (red). Scale bars, 50 μm. (**L**) Percentage of pro-Sftpc^–^cells in wildtype clones derived from slower-cycling (blue) and faster-cycling (purple) AT2 subsets in *Confetti* and *Red2Kras* lungs. Data are represented as mean ± SEM. *p* values given in the panel, paired Student’s t-tests. For (A)–(C), (E), and (F), *n* ≥ 3 mice per timepoint (total numbers of clones and mice analyzed given in Table S1). (**M**) Schematic summarizing oncogene-induced effects on wildtype AT2 behavior proximate and remote to mutant clones.

To establish whether oncogene-induced proliferation occurred in conjunction with biases in AT2 fate, we examined the composition of wildtype clones, revealing a significant increase in pro-Sftpc^–^YFP^+^ cells in *Red2Kras* lungs relative to *Confetti* controls (Figures 6A–6D and Mendeley Data Figure 1). In contrast to wildtype expansion, loss of pro-Sftpc was independent of proximity to mutant clones (up to a 300 μm resolution) (Figures 6E, 6F, and S6I), suggesting that alternate mechanisms control self-renewal versus differentiation. The transcriptomic profiles of *Red2Kras* YFP^+^ cells corroborated this departure from AT2 fate occurring alongside an increase in cycling cells (Figures 6G and 6H). Moreover, they revealed that wildtype cells entered a regenerative program, with a Primed AT2 state emerging in *Red2Kras* YFP^+^ scRNA-seq data (Figure 6H), followed by increased quantities of Krt8^+^ and Ager^+^ YFP^+^ cells detected via immunofluorescence at later timepoints (Figures 6I–6K). Interestingly, the fraction of pro-Sftpc^–^cells comprising faster-cycling YFP^+^ clones tended towards the proportion of AT1 cells typically present within homeostatic alveolar units^28^ (Figures 6A–6C), reflecting an attempt to restore a tissue-wide fate balance. However, unlike our *Confetti* data, faster-cycling *Red2Kras* YFP^+^ cells displayed greater differentiation capacity than slower-cycling cells (Figure 6L), reminiscent of the heightened regenerative potential associated with *Axin2*^+^/*Il1r1*^+^ AT2 subsets after lung injury.^9–11^

Next, we used CellChat^29^ to explore mutant-wildtype crosstalk networks underpinning accelerated *Red2Kras* wildtype dynamics. These analyses revealed a notable increase in the number and strength of bidirectional RFP-YFP interactions in *Red2Kras* lungs (Figures S6J–S6N). Specifically, we identified several pathways that were temporally upregulated in the *Red2Kras* YFP^+^ population, including Wnt, Notch, Spp1, Fgf, and Bmp (Figures S6O and S6P), all of which have previously been shown to modulate AT2 fate after injury^3,10,30,31^. Since *Spp1* and *Dlk1* were detected in mutant cells, as well as enriched within these networks (Figures 3C and S6P), we assessed whether they mediate the oncogene-induced effects on wildtype AT2 behavior. Treatment with either Dlk-1 or Spp1 enhanced wildtype organoid formation efficiency and resulted in significantly larger organoids that grew to a similar extent as those cultured alongside mutant RFP^+^ organoids (Figures S6Q–S6S). However, the addition of both ligands simultaneously counterintuitively decreased this effect on organoid size, implying that only one of these pathways may underlie the effect on wildtype AT2 dynamics. To identify which, we quantified Dlk-1 and Spp1 expression within the mutant organoids from which they are secreted. These analyses revealed a significant rise in *Spp1* expression, whilst *Dlk1* was elevated to a lesser extent (Figures S6T and S6U), implicating mutant-secreted Spp1 as a likely candidate driving enhanced wildtype AT2 expansion in oncogenic tissue.

### Signaling through *Il1r1* is required for mutant expansion and cellular reprogramming

**Figure 7.**
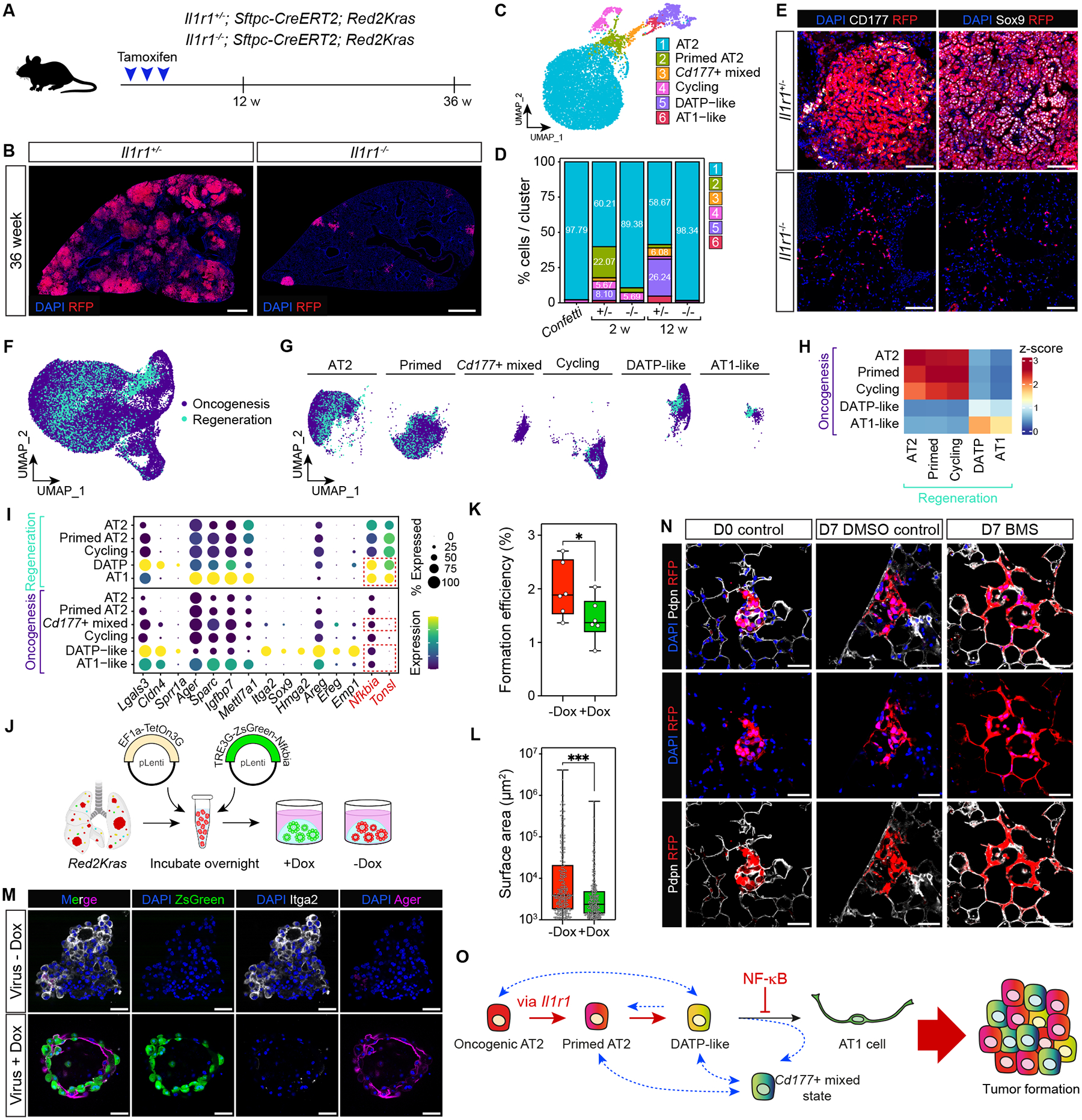
Sustained NF-κB activation distinguishes oncogenesis from regeneration. (**A**) Experimental design for lineage tracing *Il1r1*^*loxP/loxP*^*;Sftpc-CreERT2;Red2Kras* mice. (**B**) Tile scans acquired across 36-week lung lobe cross-sections from *Il1r1*^+/−^control (left) and *Il1r1*^*−/−*^(right) *Red2Kras* mice. DAPI (blue), RFP (red). Scale bars, 1000 μm. Images representative of *n* = 3 *Il1r1*^+/−^and *n* = 5 *Il1r1*^*−/−*^mice. (**C**) UMAP visualization of 9,211 epithelial cells from 2- and 12-week *Il1r1*^+/−^and *Il1r1*^*−/−*^mice, segregating into equivalent states as Figure 3B. (**D**) Percentage of RFP^+^ cells occupying each state per sample. Percentages greater than 5% are given within the corresponding bars in the plot. (**E**) Confocal images showing exclusive CD177 (white, left panels) and Sox9 (white, right panels) expression in 36-week *Il1r1*^+/−^control (top) and not *Il1r1*^*−/−*^(bottom) *Red2Kras* lungs. Images representative of *n* = 3 mice. DAPI (blue), RFP (red). Scale bars, 100 μm. (**F**) Localization of regeneration^11^ and oncogenesis (Figure 3) datasets on the integrated UMAP. Cells colored according to dataset of origin. (**G**) UMAP localization of datasets in (F), split by cellular state. (**H**) Fisher transformed z-scores highlighting the correlation between cell state signatures from datasets in (F). (**I**) Key differentially expressed genes distinguishing oncogenic DATP-like cells from regenerative DATPs. Each dot is colored according to expression levels and sized according to the proportion of cells expressing that marker. Genes highlighted in red (*Nfkbia* and *Tonsl*) are NF-κB negative regulators upregulated during AT2-to-AT1 differentiation. (**J**) Experimental design for overexpressing *Nfkbia* in mutant RFP^+^ organoids using a doxycycline-inducible ZsGreen lentiviral reporter system. (**K**) Organoid formation efficiency of mutant cells transduced as in (J) and cultured with (green) or without (red) doxycycline. Each dot represents 1 independent experiment. (**L**) 2D surface area of organoids in (K). Each dot represents 1 organoid, with 50 organoids quantified per experiment. For (K) and (L), *n* = 6 independent experiments. Centre line, median; box, interquartile range; whiskers, range. *p < 0.05; ***p < 0.001, paired Student’s t-test. (**M**) Representative confocal images showing acquisition of Ager (magenta) in transduced doxycycline-treated ZsGreen^+^ organoids. DAPI (blue), ZsGreen (green), Itga2 (white). Scale bars, 50 μm. (**N**) Representative immunostaining of AT1 marker, Pdpn (white) in 2-week precision-cut *Red2Kras* lung slices either fixed immediately (D0 control) or cultured *in vitro* and treated with DMSO (control) or BMS-345541. DAPI (blue), RFP (red). Scale bars, 50 μm. (**O**) Proposed model of tumor initiation. Signaling through *Il1r1* initiates the reprogramming cascade while sustained downstream NF-κB activation prevents AT1 differentiation and triggers acquisition of a mutant-specific *Cd177*^+^ mixed state followed by reversible state transitions that promote tumor growth.

Having characterized AT2 fate dynamics associated with tumorigenesis, we focused on uncovering the regulatory mechanisms directing their trajectory. Previously, our group established that *Il1r1* signaling is necessary for initiating AT2-driven alveolar repair.^11^ Considering the resemblance between oncogenesis and regeneration as well as the overlap between faster-cycling and *Il1r1*^+^ AT2 cells, we questioned whether this pathway might similarly mediate tumor initiation. To test this hypothesis, we performed *Il1r1* loss-of-function studies using *Il1r1*^*loxP/loxP*^;*Sftpc-CreERT2;Red2Kras* mice (Figure 7A), revealing a marked reduction in *Il1r1*-deficient mutant clone expansion persisting over the 36-week tracing period (Figures 7B and S7A). Subsequent scRNA-seq analyses confirmed that *Il1r1*^+/−^ heterozygous mutant cells expanded via a similar path to *Red2Kras* RFP^+^ cells, producing DATP-like and *Cd177*^+^ mixed states (Figures 7C, 7D, S7B, and S7C). Despite a minor transient increase in Primed AT2 and Cycling cells, no evidence of cellular reprogramming was detected either in 12 or 36-week *Il1r1*-deficient mutant populations (Figures 7D, 7E, and S7D). Altogether, these data indicate that *Il1r1* signaling is essential for driving tumor formation via mutant AT2 cellular reprogramming.

### Temporal NF-κB inhibition distinguishes alveolar regeneration from oncogenesis

Given the conserved requirement for *Il1r1* signaling in regeneration and tumorigenesis, we sought to pinpoint the stage at which these processes diverge, reasoning that this could represent a key target for therapeutic intervention. To this end, we integrated our *Red2Kras* transcriptomic data with our previously published scRNA-seq dataset obtained following bleomycin injury^11^ (Figures 7F–7I and S7E). By examining the correlation between their respective gene signatures, we identified prominent differences between regeneration-associated DATP and oncogenic DATP-like states (Figures 7G, 7H, and S7F), with the former bearing greater resemblance to primitive oncogenic AT1-like cells than to DATP-like cells themselves (Figure 7I). Meanwhile, oncogenic DATP-like cells uniquely expressed the developmental regulator *Hmga2*, as well as Egfr ligands, *Areg* and *Ereg* (Figure S7F). Crucially, we also detected a stark contrast in NF-κB signaling, the primary downstream target of *Il1r1*, between both datasets, with NF-κB negative regulators, *Nfkbia* and *Tonsl*, enriched in regeneration-associated DATP and AT1 cells but notably absent from oncogenic DATP-like and *Cd177*^+^ mixed states (Figures 7I and S7F). These findings suggested that temporal NF-κB inhibition may be involved in acquisition of AT1 identity.

Therefore, we utilized a doxycycline-inducible ZsGreen reporter to assess whether endogenous upregulation of *Nfkbia* permitted mutant cells to differentiate (Figures 7J and S7G). Overexpression of *Nfkbia* in mutant RFP^+^ cells resulted in significantly fewer organoids, all of which were smaller in size than controls (Figures 7K, 7L, and S7H), supporting the notion that NF-κB activation is required for oncogenic expansion. *Nfkbia*-expressing ZsGreen^+^ mutant organoids also contained fewer Itga2^+^ cells, but more Ager^+^ cells (Figure 7M), consistent with a role for NF-κB inhibition in coordinating the DATP-to-AT1 transition. Exogenous NF-κB blockade via BMS-345541 (BMS), a selective allosteric inhibitor of IkB kinase, similarly generated smaller mutant organoids that expressed increased levels of Ager but contained significantly fewer CD177^+^, Itga2^+^, and Sox9^+^ cells relative to DMSO-treated controls (Figures S7I–S7Q). Moreover, when mutant clones were treated with BMS *in situ* using precision cut lung slice (PCLS) *ex vivo* cultures, we discovered a remarkable change in clone morphology, where individual RFP^+^ cells flattened and began to phenotypically resemble AT1 cells, upregulating Pdpn (Figure 7N). Collectively, our findings suggest that NF-κB blockade is sufficient to shift mutant-specific cell states toward a differentiated AT1 phenotype.

Given these striking results, we assessed whether sustained NF-κB activation likewise induced mutant-specific cell state signatures in wildtype AT2 cells by treating them *in vitro* with IL-1β to constitutively activate downstream NF-κB. In accordance with our prior work, continual IL-1β treatment significantly increased the size of wildtype organoids and suppressed AT1 differentiation.^11^ In addition, whilst CD177 expression was undetectable in wildtype control organoids, those treated with IL-1β became enriched in CD177^+^ cells, indicative of a transition towards mutant-associated states (Figures S7R–S7V). Administering BMS in conjunction with IL-1β from day 7 of culture was sufficient to completely reverse this phenotype, resulting in significantly smaller organoids that contained Pdpn^+^ but not CD177^+^ cells (Figures S7R–S7V).

Interestingly, our earlier experiments suggested that accelerated wildtype AT2 expansion in oncogenic tissue is driven by Spp1 (Figures S6Q–S6S), which similarly functions via downstream NF-κB activation. Moreover, we found that wildtype AT2-derived organoids cultured with mutant organoids significantly upregulated *Nfkbia* compared to those cultured alone (Figure S6V), signifying that, unlike their mutant counterparts, wildtype cells retain an intact NF-κB negative feedback loop. Taken together, these results suggest that mutant cells utilize reciprocal regenerative mechanisms to influence the fate of their neighbors.

## DISCUSSION

Here, we conducted large-scale *in vivo* quantitative analyses of mouse AT2 dynamics during homeostasis and tumorigenesis to uncover regulatory mechanisms governing LUAD evolution. Our data reveal that AT2 cells operate within a conserved two-population model, with a faster-cycling subset characterized by enhanced tumorigenic potential. We found that interleukin-1 signaling orchestrated this oncogenic response by reprogramming mutant cells into regenerative-like states, while sustained downstream NF-κB activation enabled them to bypass differentiation in favor of tumor growth (Figure 7O). These events, in turn, elicited a widespread regenerative-like response in surrounding wildtype AT2 cells, which attempted to re-establish a tissue-wide AT2:AT1 fate balance.

Our work adds to a growing body of evidence detailing functional heterogeneity within the AT2 compartment^9–11^ and illustrates how oncogenic activation exploits these differences to induce rapid tumor growth. While we cannot conclusively exclude the possibility that slower- and faster-cycling populations belong to a single stem cell hierarchy (Methods S1), the dynamics exhibited by each fraction showed no evidence of interconversion, making this scenario unlikely. Instead, our data favor a model in which the two subsets function independently, with fate balance enforced through neutral competition between adjacent clones, analogous to homeostatic mechanisms in other epithelia.^32,33^ Since both slower- and faster-cycling clones exhibited differentiation capacity, it is plausible that their respective AT1 progeny correspond to molecularly or functionally distinct subtypes.^34^ Nevertheless, differentiation events were rarer than anticipated given the fraction of AT1 cells comprising the alveolar epithelium^28^, suggesting that AT1 cells have a prolonged half-life, as described for ciliated cells in the airway.^35,36^

Notably, we found that co-option of a regenerative program initiated through *Il1r1* signaling^11^ was an essential feature of mutant AT2-driven clonal expansion, providing further functional evidence of a link between faster-cycling and *Il1r1*^+^ AT2 fractions. Given that IL-1β has similarly been implicated in *Egfr*-driven LUAD initiation following air pollutant exposure^37^, our data imply that an *Il1r1*-dependent mechanism may play a unified role in initiating lung tumorigenesis, even without an existing permissive inflammatory microenvironment. Despite these parallels with regeneration, mutant cells fail to differentiate, instead transitioning into an aberrant *Cd177*^+^ mixed state. We found that reversible transitions between mutant states were hallmark of this lineage infidelity, allowing initial heterogeneity in tumor cell dynamics to stabilize, resulting in diverse, yet equipotent, mutant states with similar proliferative potential. Our model suggests that stochastic variations in individual cell cycling rates within each state then produce the varied clone sizes and transcriptional signatures seen across the tissue. In contrast to these findings, several recent reports predicted the presence of an Itga2^+^ “high plasticity cell state”^17,18^ and observed positive selection of aggressive metastatic subclones^20^ in AT2-derived tumors following concomitant *Kras* activation and *Trp53* deletion. These conflicting results suggest that further oncogenic hits may disrupt the balance in cell dynamics we observed among *Kras*-mutant states with deleterious consequences. Future research into the mechanisms facilitating this dynamic shift may thus prove invaluable for advancing our understanding of how intra-tumoral heterogeneity is acquired.

We find that endogenous upregulation of NF-κB inhibitors in DATPs forms an autoregulatory negative feedback loop that governs AT1 differentiation and, importantly, distinguishes regeneration from oncogenesis. Indeed, *Nfkbia* overexpression was sufficient to block mutant expansion and promote AT1-like differentiation, while continual NF-κB activation induced mutant-specific signatures in wildtype cells. Although NF-κB has previously been linked to LUAD progression^38,39^, our findings reveal its role in modulating AT2 fate, illustrating how this lineage-specific feedback circuitry is manipulated for cancer initiation, with broader relevance for diseases like IPF that similarly arise from impaired AT2 differentiation. Based on our data, we propose that *Kras*-mutant cells remodel their microenvironment to trigger secretion of inflammatory signals that initiate *Il1r1*-mediated NF-κB signaling, with NF-κB and Ras-Raf crosstalk^40^ contributing to sustained NF-κB activation in the long term.

Our results show that these mutant-specific events directly affect the fate of tissue-sharing wildtype AT2 cells, offering a potential explanation for the phenotypic changes observed in non-malignant regions surrounding human LUAD tumors.^22^ While we do not yet know if these biases in wildtype AT2 dynamics promote tumor progression, recent reports have shown that modulating mutant-wildtype interactions can serve as a viable cancer preventative strategy, underscoring the need for further exploration.^41–44^ Moreover, we discovered that small remote mutant clones often expressed Cl-Casp3, suggesting that they may be actively eliminated by fitter wildtype neighbors, as occurs in other tissues.^41,42^ Interestingly, while wildtype AT2 proliferation and differentiation were both enhanced in oncogenic tissue, we discovered a surprising contrast in their proximity-dependence, indicating a possible decoupling in their regulation during tumorigenesis. Future studies investigating the existence of such decoupling mechanisms could thus prove vital for determining whether these processes can be harnessed to promote wildtype fitness for therapeutic gain.

Our findings shed light on the dynamic trajectories underpinning LUAD evolution and demonstrate how feedback loops coordinating lung regeneration are subverted to drive oncogenesis. Moving forward, the experimental and mathematical framework presented here could pave the way for developing new therapeutic strategies and delineating comparative mechanisms governing lineage infidelity across a range of tissues.

### Limitations of the study

Although we have provided comprehensive quantitative insights into the mechanisms biasing AT2 dynamics toward tumorigenesis, several limitations remain. Firstly, future studies validating the effects of NF-κB inhibition on mutant clones *in vivo* and investigating the mechanism by which DATPs acquire expression of endogenous NF-κB negative regulators will be necessary to inform the clinical utility of our findings. Second, although we observed differing dynamics in wildtype AT2 cells situated proximate and remote to mutant clones, further exploration is needed to delineate how these opposing behaviors are regulated. Third, the model we propose here is predicated on quantitative insights obtained from analyzing expanding labelled clones using low levels of tamoxifen induction. As such, we cannot exclude the possibility of additional non-proliferative or rare AT2 subsets existing within the tissue, which may remain undetected in our dataset.

## RESOURCE AVAILABILITY

### Lead contact

Further information and requests for resources should be directed to and will be fulfilled by the lead contact, Joo-Hyeon Lee (leej49@mskcc.org)

### Materials availability

This study did not generate any unique reagents.

### Data and code availability

scRNA-seq data generated in this study have been deposited at NCBI’s Gene Expression Omnibus under accession number GSE247505. Accession numbers for existing publicly available data re-analyzed here are listed in the Key Resources Table. Extracted clonal properties and scripts for two-compartment model simulations have been deposited in Zenodo (available at https://doi.org/10.5281/zenodo.14673088). Figures deposited in Mendeley Data are available at https://doi.org/10.17632/ss6pb96pty.1. Additional information required to reanalyze the data reported here is available from the lead contact upon request.

## STAR METHODS

### EXPERIMENTAL MODEL AND STUDY PARTICIPANT DETAILS

#### Mice

Mouse experiments were conducted in accordance with UK Home Office Project Licenses PC7F8AE82 and PP3176550, regulated under the Animals (Scientific Procedures) Act 1986 Amendment Regulations 2012 following ethical review by the University of Cambridge. Mice were bred and maintained in specific pathogen-free conditions at the Gurdon Institute, University of Cambridge and housed with a 12-hour (h) day/night cycle, receiving food and water *ad libitum*. Inducible mouse strains *Sftpc-CreERT2* (RRID:IMSR_JAX:028054)^43^, *R26R-Confetti* (RRID: IMSR_JAX:013731)^33^, *R26R-ZsGreen* (RRID:IMSR_JAX:007906), and *Il1r1*^*loxP/loxP*^ (RRID:IMSR_JAX:028398)^44^ have been described and were obtained from The Jackson Laboratory. *Il1r1-CreERT2* and *Red2Kras* mice were generated inhouse as previously described.^11,25^ All transgenic mouse strains were maintained on a C57BL/6Brd-Tyrc-Brd mixed background, with experimental *Sftpc-CreERT2;Red2Kras;Il1r1*^*loxP/loxP*^ cohorts generated by backcrossing to *Il1r1*^*loxP/loxP*^ mice on a C57BL/6 background. In addition, we used previously described NSG mice^45^ for *in vivo* cell transplantation studies, which were obtained from The Jackson Laboratory (RRID:IMSR_JAX:005557). Both male and female age-matched 8-week-old adult mice were used for all experiments and randomly assigned to experimental groups with no gender-specific differences observed.

#### Maintenance of primary cultures

##### 3D lung organoid cultures

Primary AT2-derived organoids were plated in Matrigel (Corning) domes and submerged with 500 μL complete Wnt culture medium, containing 1X B-27 supplement (Gibco), 100 ng/mL each of recombinant FGF7 (Peprotech), FGF10 (Peprotech), and Noggin (Peprotech), 50 ng/mL recombinant EGF (Life Technologies), 1 mM N-acetyl cysteine, 10 mM nicotinamide, and 1X Chiron (CHIR99021, Tocris Bioscience). Organoids were maintained at 37 °C under 5% CO2, and the media was changed every other day for the duration of culture. 10 μM ROCK inhibitor Y-27632 (Cambridge Bioscience) was added to the media for the first 48 h of culture to aid cell survival post-sorting.

##### Precision cut lung slice (PCLS) ex vivo cultures

PCLS sections were each placed into a single well of a 24-well plate and cultured *in vitro* in PCLS culture media containing DMEM/F-12 supplemented with 10 mM HEPES buffer, 1% penicillin/streptomycin, 1% L-glutamine, 10% FBS, and 1 mM 8-cAMP in the presence of either DMSO (control wells) or 10 μM BMS-345541. Cultures were maintained for 7 days at 37 °C under 5% CO2 and the media changed and treatments replenished every other day for the duration of culture.

### METHOD DETAILS

#### Mouse procedures

##### Tamoxifen administration

Tamoxifen (Sigma) was dissolved in corn oil (Sigma) overnight at 40 °C to generate either 2 mg/mL or 20 mg/mL stock solutions. Aliquots were heated to 50 °C and vortexed thoroughly before use. Mice were weighed and administered with either tamoxifen (0.02 mg/gbw for clonal *Confetti* experiments, 0.1 mg/gbw for clonal *Red2Kras* experiments, 5 × 0.2 mg/gbw administered every other day for clonal *Il1r1-CreERT2* experiments, and 3 × 0.2 mg/gbw administered every other day for all other experiments) or corn oil (10 μL/gbw) via oral gavage and collected at the timepoints indicated. To capture initial AT2 behavior as accurately as possible following oncogenic activation, *Red2Kras* mice were also collected at the 4-day timepoint before completion of the tamoxifen washout period. Where possible, littermates were used across multiple timepoints to control for litter-to-litter variation in long-term lineage tracing analyses.

##### EdU administration

EdU (Invitrogen) was dissolved in PBS (Sigma) at room temperature (RT) to generate a 1 mg/mL stock solution. Aliquots were heated to 37 °C and vortexed thoroughly prior to administration via intraperitoneal (IP) injection at a dose of 100 μg per mouse. The EdU pulse was consistently initiated at 10:00 am across all cohorts, with a subsequent chase period of either 2 h or 2 weeks post-injection.

##### In vivo cell transplantation

Mutant RFP^+^ cells occupying either the DATP-like, *Cd177*^+^ mixed, DN, or DP state were isolated via fluorescence-activated cell sorting (FACS) from *Red2Kras* mice 3–6 weeks post-induction and orthotopically transplanted into adult NSG mice via intratracheal injection together with supportive RFP^–^tumor stromal cells (CD31^–^CD45^–^EpCAM^–^) mixed in a 1:1 ratio to aid cell engraftment. Engrafted RFP^+^ cell populations were then isolated and analyzed via flow cytometry 2 weeks later.

#### Tissue harvest and fixation

Mice were culled by cervical dislocation at the timepoints indicated and the lungs cleared of blood through perfusion with 10 mL of cold PBS. For fixation, lungs were slowly inflated by intratracheal injection of 2 mL 4% paraformaldehyde (PFA, Thermo Fisher Scientific) in PBS before being dissected out from the thoracic cavity and immersed in 4% PFA for 2–4 h at 4 °C. Lungs were washed three times with PBS at RT for 20 min followed by a final PBS wash overnight at 4 °C. In preparation for sectioning, individual lung lobes were separated and successively submerged in 15% then 20% sucrose (Sigma) solutions in PBS at RT for 1 h each then left in 30% sucrose at 4 °C overnight. Lobes from each lung were cut into smaller pieces with a scalpel and embedded in single cryo-embedding molds containing 100% Optimal Cutting Temperature (O.C.T.) compound (VWR), immediately frozen on dry ice, and stored at −80 °C for cryo-sectioning.

#### Immunofluorescent staining of thick sections

For clonal analyses, either one (*Confetti* lungs) or three consecutive (*Red2Kras* lungs) thick 150 μm tissue sections were acquired from each cryo-block using a Cryostat (Leica). Samples were permeabilized with 0.3% Triton X-100 (Sigma) for 15 min at RT then washed three times with PBS and incubated overnight at 4 °C in blocking and permeabilization buffer, comprised of 5% DMSO (Santa Cruz Biotechnology) and 0.5% Triton X-100, with 5% normal donkey serum (NDS, Jackson ImmunoResearch Labs). Primary antibodies (Table S2) diluted in blocking and permeabilization buffer containing 5% NDS were added to each well and incubated for 72 h at 4 °C. Samples were then washed five times with wash buffer, consisting of 1% DMSO and 0.5% Triton X-100 over a 12 h period at RT. Secondary antibodies (Table S2) diluted in wash buffer with 5% NDS were added to each sample and incubated for 72 h at 4 °C. Sections were washed twice with wash buffer and three times with PBS over a 12 h period at RT before being stained with 1 μg/mL of 4’,6-diamidino-2-phenylindole (DAPI, Sigma) diluted in PBS for 20 min followed by three 30 min PBS washes, all at RT. For all incubation periods, samples were placed on a rocking shaker to ensure even distribution and penetration of staining. To mount samples, sections of each lobe were transferred over to individual microscope slides (VWR or Leica) where they were contained within 0.2 mm i-Spacers (SunJin Lab) overlaid with 0.05 mm double-sided stickers (SunJin Lab). For optical tissue clearing, sections were coated with RapiClear 1.52 (SunJin Lab), enclosed with 24×40 mm glass coverslips (VWR), and sealed with nail polish for confocal imaging.

For EdU quantification, single thick 150 μm tissue sections were acquired from treated *Sftpc-CreERT2; Red2Kras* lungs and staining performed as described above with the Click-iT^™^ Plus EdU Alexa Fluor^™^ 647 imaging kit (Invitrogen) used as per the manufacturer’s instructions after primary antibody incubation. Following incubation with the Click-iT^™^ reaction cocktail for 30 min at RT, samples were washed three times with PBS before proceeding with secondary antibody staining.

#### Immunofluorescent staining of thin sections

Thin 15 μm cryosections were collected directly onto glass microscope slides and placed into a humidified chamber. Here, individual sections were circled using a hydrophobic PAP pen (Sigma) and permeabilized with 0.3% Triton X-100 for 15 min, followed by blocking with 0.2% Triton X-100 in PBS containing 5% NDS for 1 h at RT. Primary antibodies (Table S2) were diluted in the same buffer and incubated overnight at 4 °C. Sections were washed three times with 0.2% Tween-20 (Sigma) for 5 min each at RT before incubating with secondary antibodies (Table S2) diluted in PBS with 5% NDS for 2 h at RT. Sections were washed three times with 0.2% Tween-20 for 5 min each and the nuclei stained with 0.5 μg/mL of DAPI in PBS for 10 min at RT. Following three final PBS washes, all liquid was removed, and samples mounted with RapiClear 1.52, enclosed with a 24×50 mm glass coverslips (VWR), and sealed with nail polish.

#### Confocal imaging

All IF images were acquired with either a Leica DMi8 SP8 or a Leica STELLARIS 8 white light laser inverted confocal microscope. Standard laser configurations were used in all cases, with the software set to bidirectional scanning, a pinhole of 1 Airy unit, a zoom factor of 1, and a speed of 400 Hz.

For large-scale clonal analyses, tiled z-stack images were taken across the entire cross-sectional area of individual lung lobe pieces through the full 150 μm depth using the SP8 20x multi-immersion oil objective. In *Red2Kras* samples, separate tiled z-stacks were acquired both across and through each of the three consecutive thick tissue sections and later merged in post-processing (Figure 2B). A minimum of three lung lobe pieces was sampled per mouse to capture a statistical ensemble of clones throughout the tissue, with a minimum of *n* = 3 mice imaged per timepoint. Confocal settings were optimized for automated detection of clonal size and composition while reducing image acquisition time as far as possible, with a step size of 5 μm and a resolution of 512×512 pixels found to be suitable. All tiled z-stack images were stitched together using inbuilt Leica LAS X software before being exported for downstream analyses.

To perform manual quantification of EdU^+^ cells within oncogenic clones, z-stack images were taken through multiple individual RFP^+^ clones in thick 150 μm tissue sections with a step size of 3 μm and a spatial resolution of 512×512 pixels. Quantification of other markers expressed across the lineage-labelled population was conducted by acquiring ten independent regions approximately 1 mm^2^ in area (~9 tiled frames), sampled from all five lobes of the lung under the 40x oil objective of the STELLARIS 8.

Representative 3D reconstructions of individual clones in *Confetti* and *Red2Kras* samples were obtained by capturing z-stack images through the full depth of single clones using a step size of 0.5 μm, a resolution of 1024×1024 pixels and a line average of 3 and performing 3D reconstruction with Imaris x64 9.4.10 (BitPlane) software. Representative frames for all samples were taken using either the SP8 or STELLARIS 8 40x oil objectives with a spatial resolution of 1024×1024 pixels and a line average of 3.

#### Semi-automated clonal assignment and 3D reconstruction

##### Alignment and merging of consecutive tissue sections

Tile scans acquired through the full depth of three consecutive thick 150 μm *Red2Kras* tissue sections required alignment and merging before semi-automated image segmentation and clonal assignment could be performed. This was achieved during post-processing using MATLAB (MathWorks) software. First, tiled z-stacks for each successive tissue section were aligned in the x-y plane using a geometric transformation, where corresponding landmarks were manually identified and matched across consecutive sections from the same lobe piece, facilitating image translation. Individual sections were then shifted in the z-plane along the transversal direction so that clones which were bisected during sectioning could be reconstructed (Figure 2B). In this way, the three tissue sections from each lobe were merged into cohesive 3D z-stacks, matching single section data obtained from *Confetti* samples.

##### Cluster segmentation and clonal assignment

To reconstruct individual clones from either single section tiled z-stacks (*Confetti* samples) or merged multi-section tiled z-stacks (*Red2Kras* samples), a fluorescence intensity-based approach was implemented in MATLAB, as previously described.^23^ Here, clusters of cells marked by the same fluorophore were initially detected, with each cluster defined as a collection of pixels lying no more than 5 μm away from other pixels of the same color. Although four labelling outcomes are technically possible in *Confetti* and *Red2Kras* systems, we focused solely on quantifying RFP^+^ and YFP^+^ cells, which occurred most abundantly in our system and were both cytoplasmically localized. After cluster identification, the characteristic distance below which two clusters were likely to belong to the same clone was determined by computing the pair correlation function of clusters marked by the same fluorophore. This analysis revealed a characteristic distance of approximately 50 μm, which was largely consistent across datasets and timepoints and thus used for all clonal assignment, with cell clusters labelled with the same fluorophore within a 50 μm radius of one another considered as part of the same clone.

Following clonal assignment, datasets were visually inspected to evaluate the morphology of reconstructed clones and identify potential artefacts, including evident clone mergers. In *Confetti* and *Red2Kras* YFP^+^ samples, an average of 1 clone per sample was considered an artefact or merger and discarded from further analyses. For the oncogenic RFP^+^ compartment in *Red2Kras* samples, clones organized preferentially as spheroidal structures, facilitating easy identification of merger events. In these datasets, between 5 and 12 clones per sample were considered artefacts, corresponding to 0.4–2% of the total number of clones in each dataset. Datasets where labelling was deemed to be below the threshold at which clonal reconstruction could be reliably performed were excluded from analyses.

Once clonal identity was confirmed, constituent cell numbers were estimated by normalizing clone volume, in pixels, against the typical size of a single cell in each sample, computed by averaging the size of all clusters of pixels smaller than 350 pixels or ~16 μm in diameter. In this way, slight differences in fluorophore intensity across samples imaged on different days could be accounted for.

In all samples, a high fraction of clones composed of only a single cell (singlets) were detected. As these single-cell clones could either represent proliferative or non-proliferative populations, analysis of clonal dynamics was performed by focusing solely on the definitive proliferative clones, composed of 2 or more cells.

#### Live tissue harvest and digestion

For isolation of live lineage-labelled cells, mice were culled via cervical dislocation and lungs cleared of blood through perfusion as before. Lungs were carefully dissected out of the thoracic cavity with the tracheae still intact and placed into a petri dish on ice, where they were inflated with 2 mL of dispase solution (Corning) via intratracheal injection. Following a 2 min incubation period on ice, individual lobes were separated from each lung and minced into small pieces (<1 mm in size) using extra narrow 10.5 cm scissors (InterFocus). Cells were washed down to the bottom of each tube using 3 mL of cold PBS and 60 μL of 100 mg/mL collagenase/dispase solution (Sigma) was added per tube. Samples were placed into a shaking incubator at 37 °C set to 180 revolutions per minute (rpm) for 45 mins and 7.5 μL of 1% DNase I (Sigma) was added to each sample in the final 10 min of incubation. Dissociated tissue cell suspensions were filtered sequentially through 100 μm and 40 μm cell strainers and washed with 3 mL of 10% fetal bovine serum (FBS, Pan-Biotech) in PBS (PF10) to collect remaining cells. Tubes were centrifuged at 800 rpm for 5 min at 4 °C, after which the supernatant was aspirated. Cell pellets were resuspended in 1 mL of red blood cell lysis buffer, prepared inhouse with 150 mM NH_4_Cl and 10 mM KHCO_3_ in distilled H_2_O. Following a 90 second incubation at RT, cell lysis was inactivated with 6 mL of Dulbecco’s Modified Eagle Medium Nutrient Mixture F-12 (DMEM/F-12, Invitrogen), and 500 μL of FBS then added slowly to the bottom of each tube to collect live cells. After a second centrifugation step at 800 rpm for 5 min at 4 °C, the supernatant was aspirated, and cell pellets resuspended in PF10 and placed into separate Eppendorf tubes ready for antibody staining, with a small fraction of cells set aside for unstained and single-stained antibody controls.

#### Fluorescence-activated cell sorting (FACS)

Depending on the populations to be isolated, relevant FACS antibodies (Table S2) were added to each sample at a 1 in 200 dilution in PF10 and incubated on ice for 20 min. Following incubation, cells were spun down at 4 °C and the supernatant discarded. All cell pellets were resuspended in PF10 and filtered through a 35 μm cell strainer (VWR) into polypropylene FACS tubes (Corning). Samples were sorted using a BD Influx^™^ cell sorter through a 100 μm nozzle, with individual cell populations collected into chilled Eppendorf tubes containing 500 μL FBS. Analysis of flow cytometry data was performed using FlowJo v10.9.0.

#### Single-cell transcriptomic profiling

##### Library preparation and sequencing

To enable sufficient cells to be isolated for single-cell RNA sequencing (scRNA-seq) and ensure that the majority of mutant and wildtype cells were in close contact with one another *in vivo*, *Sftpc-CreERT2; R26R-Confetti*, *Sftpc-CreERT2; Red2Kras* and *Sftpc-CreERT2; Red2Kras; Il1r1*^*loxP/loxP*^ mice used for scRNA-seq experiments were induced with a bulk dosage of tamoxifen (3 doses of 0.2 mg/gbw administered every other day). Lungs were harvested and dissociated as described above and single-cell suspensions containing EpCAM^+^ cells that were either RFP^+^ or YFP^+^ were then isolated via FACS. In all cases, a minimum of two lungs were pooled together for each sample to minimize variability and enable isolation of sufficient cells. For comparative analyses between *Red2Kras* and *Confetti* lungs, both RFP^+^ and YFP^+^ cells were isolated 4 days (*Red2Kras* only) and 2 weeks (*Red2Kras* and *Confetti*) post-induction, with a total of 13 libraries generated across two days (1 x *Confetti*, 2 × 4 day, and 2 × 2 week *Red2Kras*). For *Il1r1*^*loxP/loxP*^ studies, RFP^+^ cells were isolated 2- and 12-weeks post-induction, with *Confetti* RFP^+^ cells serving as a homeostatic control. In each case, cell suspensions were spun down, counted, and resuspended in 0.04% bovine serum albumin (BSA) in PBS to achieve a desired cell concentration of ~8,000 cells in a final volume of 45 μL. Single-cell 3′ RNA-seq libraries were generated according to manufacturer’s instructions (Chromium Single Cell 3′ Reagent v3 Chemistry Kit, 10x Genomics) and cDNA quality was assessed using the Agilent TapeStation 4200. Libraries were then multiplexed and sequenced to a minimum depth of ~20,000 reads per cell using Illumina Novaseq 6000.

##### Read alignment

Raw FASTQ files containing droplet-based sequencing data were pre-processed in CellRanger v3.1.0. Reads were aligned to the Ensembl Mus musculus GRCm38 reference genome, empty droplets were filtered out and the number of unique molecular identifiers (UMIs) mapped to each protein-coding gene were quantified to generate a final count matrix with feature and barcode annotations.

##### Quality control

Downstream analyses of mapped reads were performed using Seurat package v4.3.0^47^ in R v4.2.1. Quality control metrics for individual libraries were first inspected and subsequently used to define thresholds for filtering out low quality cells, with custom cut-offs of >15% mitochondrial genes, <1000 detected genes, and >50,000 UMIs used in all cases. Mitochondrial genes, as well as any genes expressed in fewer than 3 cells, were removed from the count matrix before normalization to ensure they did not interfere with downstream clustering algorithms.

##### Dimensionality reduction and clustering

The Seurat pipeline was used for dimensionality reduction and clustering of all scRNA sequencing datasets. Data was log normalized and scaled, with the top 5,000 highly variable genes used for principal component analysis (PCA). Graph-based clustering was performed with the Louvain algorithm and the results visualized using the first two uniform manifold approximation and projection (UMAP) dimensions. Clusters containing unwanted cell types were discarded based on expression of key marker genes *Ptprc* (immune cells), *Pecam1* (endothelial cells), *Col1a1* (mesenchymal cells), and *Foxj1* (ciliated cells), alongside other canonical markers. Normalization, dimensionality reduction, and clustering was performed separately for different batches of the same sequencing run, with the filtered Seurat objects integrated for further analyses. Count matrices were then renormalized and PCA-based dimensionality reduction, clustering, and UMAP visualization were performed. Individual cell clusters were annotated based on known marker genes and comparison with recently identified cell states in a lung regeneration context.^11,26,27^ Markers for each cluster were acquired using the FindMarkers function and gene expression between clusters visualized using the DotPlot function and the ComplexHeatmap package (v.2.15.1).^48^

##### Cell-cell communication analyses

Identification of communication networks and ligand-receptor interactions between mutant and wildtype cells was performed using CellChat.^29^ Here, communication probabilities between clusters were computed based on the fully curated CellChatDB mouse database. Analyses are presented for interactions displaying a communication probability with *p* < 0.05.

##### Integration of oncogenesis and regeneration datasets

To enable direct comparison of transcriptional changes underlying cellular dynamics during regeneration and oncogenesis, our previously published alveolar regeneration dataset^11^ was integrated with our oncogenic dataset using the Seurat package following the pre-processing and filtering steps detailed above. 2,000 common anchor features between both datasets were then identified using the FindIntegrationAnchors function, with the data subsequently integrated using IntegrateData. From here, dimensionality reduction, normalization, scaling, and post-processing was performed as detailed above. The correlation matrix between the transcriptional signatures of clusters in both datasets was computed by calculating the Pearson correlation coefficient using the averaged expression of the top 1,000 differentially expressed genes. A Fisher transformation was subsequently applied to generate the corresponding z-score.

#### Generation of primary 3D organoid cultures

Organoids from mouse AT2-derived cells were established as described previously.^11,46^ Briefly, freshly sorted EpCAM^+^ lineage-labelled cells were centrifuged at 300 g for 10 min at 4 °C and resuspended in basic Wnt medium containing Advanced DMEM (Fisher Scientific) supplemented with 10 mM HEPES buffer (Invitrogen), as well as 1% penicillin/streptomycin and 1% L-glutamine (supplied by Cambridge Stem Cell Institute Tissue Culture Core Facility). Cell numbers were quantified manually using C-chip disposable hemocytometers (VWR) and cell concentrations were adjusted accordingly before centrifuging samples as before. Individual cell pellets were carefully resuspended in growth factor reduced Matrigel basement membrane matrix at a concentration of 5,000–10,000 cells per well, with each well comprising single 20 μL Matrigel domes plated either on 8-well μ-slides (ibidi) or 8-well LabTek chamber slides (ThermoFisher). Domes were left to set at 37 °C for 30 min before submerging in complete Wnt culture medium.

##### Mutant-wildtype competition organoid cultures

To study paracrine signaling between oncogene-expressing RFP^+^ and wildtype AT2 YFP^+^ cells, individual organoids were established from both compartments and cultured either in separate wells, or in the same well but in separate Matrigel domes within ibidi 8-well μ-slides. This design allowed the effects of paracrine signals to be explored without potential confounding effects arising due to space limitations, mechanical interactions, or inherent differences in proliferative dynamics. In this case, individual domes were formed from 17 μL Matrigel droplets to prevent domes fusing prior to setting.

##### Pharmacological modulation of organoid cultures

To assess whether the oncogene-induced effects on wildtype organoids could be recapitulated with mutant-secreted ligands, wildtype organoids were treated either with 100 ng/mL recombinant mouse Dlk-1 (R&D Systems) or 1 μg/mL recombinant mouse osteopontin (Spp1) (R&D Systems) or both as indicated starting from day 2 of organoid culture. Ligands were replenished with each media change and maintained for the remainder of culture, with organoids analyzed on day 14.

For pharmacological inhibition of NF-κB signaling, organoids were treated either with DMSO (vehicle control) or with 5 μM BMS-345541 (Abcam) as indicated from day 7 of organoid culture. Compounds were replenished with each media change and maintained throughout the duration of culture, with organoids imaged and fixed for analysis on day 14. To assess the impact of continual NF-κB activation, wildtype organoids were treated every other day with 20 ng/mL recombinant murine IL-1β (Peprotech) starting from day 2 of culture. Media was then supplemented with either DMSO (vehicle control) or 5 μM BMS-345541 from day 7 as indicated (IL-1β + BMS wells).

#### Lentiviral synthesis and production

pLVX-EF1a-Tet3G and pLVX-TRE3G-ZsGreen plasmids were a gift from Jonghwan Kim. The *Nfkbia* gene was cloned into the pLVX-TRE3G-ZsGreen construct using the In-Fusion HD cloning kit (Clontech) as per manufacturer’s instructions, with correct targeting confirmed via Sanger sequencing. To generate each lentivirus, the corresponding plasmid and associated lentiviral packaging vectors were transfected into HEK293T cells grown in DMEM supplemented with 10% FBS and 1% PenStrep using the TransIT^®^-LT1 Transfection Reagent (MIR2300, GeneFlow) as per manufacturer’s instructions. Lentivirus was harvested twice over 24 hours and concentrated using ultra-centrifugation at 5,000 rpm for 20 h.

#### Lentiviral transduction and organoid culture

Mutant RFP^+^ cells were transduced directly after FACS isolation by incubation with equal quantities of both pLVX-EF1a-Tet3G and pLVX-TRE3G-ZsGreen-Nfkbia viruses diluted in Advanced DMEM containing ROCK inhibitor. After a 12 h incubation step at 37 °C in 96-well round bottom plates, wells were washed twice with Advanced DMEM and the collected cells centrifuged at 300 g for 10 min. Cells were counted and resuspended in Matrigel at a concentration of 5,000–10,000 cells per 20 μL dome. From 48 h after plating, ROCK inhibitor was removed and doxycycline added to each well to a final concentration of 0.5 μg/mL, with no doxycycline added to control wells. Complete Wnt media and doxycycline was replaced every other day for the duration of the culture.

#### Immunofluorescent staining of wholemount organoids

Wholemount organoids were cultured in LabTek chamber slides and fixed in situ with 200 μL of 4% PFA for 20 min in the dark at RT. Wells were washed gently with PBS three times for 5 min each at RT, taking care not to disturb the organoid structures. For IF staining, organoids were permeabilized with 0.5% Triton X-100 in PBS for 30 min at RT followed by blocking with 0.3% Triton X-100 in PBS containing 5% NDS for 1 h at RT. Primary antibodies (Table S2) diluted in 0.2% Triton X-100 in PBS with 5% NDS were added to each well and the slides incubated at 4 °C for 48 h. Wells were washed three times with 0.2% Tween-20 in PBS and incubated with secondary antibodies (Table S2) diluted in 0.2% Triton X-100 in PBS containing 5% NDS at 4 °C for 48 h. Organoids were washed three times with 0.2% Tween-20 and stained with 1 μg/mL DAPI for 30 min at RT followed by three PBS washes. For mounting, chambers were removed from LabTek slides as per the manufacturer’s instructions and dried with tissue paper before covering with RapiClear 1.52 and enclosing with a 24×50 mm glass coverslip sealed with nail polish and left to penetrate over a 24 h period at RT before imaging.

#### Organoid imaging

Fluorescence images of live organoid cultures were acquired using a Leica DMI6000 B inverted microscope under a 2.5x objective on day 14 of culture. To capture the full well, tiled images were taken over the entire region and stitched together using inbuilt LAS X software. For IF analyses, stained wholemount organoids were imaged with a Leica STELLARIS 8 under the 40x oil objective. Representative images were acquired with a spatial resolution of 1024×1024 pixels and a line average of 3.

#### Generation of PCLS *ex vivo* cultures

To generate PCLS *ex vivo* cultures, lungs were dissected out of the thoracic cavity and slowly inflated with pre-heated 2% certified low melt agarose (Bio-Rad) through the trachea before being briefly submerged in ice-cold PBS to allow to gel. Individual lung lobes were then separated, washed with cold PBS, and embedded in cryo-embedding molds filled with 2% certified low melt agarose in preparation for sectioning. Consecutive thick 150 μm sections were acquired from 2–3 lung lobes per mouse using a vibratome (Leica VT1200) and washed three times with PCLS wash media containing DMEM/F-12 (Fisher Scientific) supplemented with 10 mM HEPES buffer, 1% penicillin/streptomycin, 1% L-glutamine, 1.25 μg/mL amphotericin B (Sigma), 100 mM 3-isobutyl-1-methylxanthine (IBMX) (Sigma), and 1 mM 8-bromoadenosine 3’−5’-cyclic monophosphate (8-cAMP) (Sigma). Red blood cell lysis buffer was added to these sections for 5 minutes, followed by three more wash steps as before to prevent further cell lysis. Each PCLS section was then placed into a single well of a 24-well plate and cultured *in vitro* in PCLS culture media. PCLS sections were fixed using 4% PFA for 30 minutes at RT on day 7 of *ex vivo* culture, washed three times with PBS, and prepared for immunofluorescent analyses using our thick section staining protocol.

#### RNA extraction and quantitative PCR (qPCR)

Cell pellets from individual populations were isolated via FACS, snap frozen on dry ice and stored at −80 °C. RNA extraction was then performed from frozen cell pellets using TRIzol^™^ Reagent (Invitrogen) as per manufacturer’s instructions. RNA concentrations were assessed using a NanoDrop^™^ microvolume UV-Vis spectrophotometer (Thermo Fisher). For DNase treatment, 1 μL of 10X DNase reaction buffer and 1 μL of amplification grade DNase I (Invitrogen) was added to each RNA sample and incubated for 15 min at RT before inactivation with 1 μL of 25 mM EDTA and heating to 65 °C for 10 min. From here, cDNA synthesis was performed using the SuperScript^™^ IV First-Strand Synthesis System (Thermo Fisher) as per manufacturer’s instructions.

All qPCR reactions were performed in MicroAmp^™^ fast optical 96-well plates (Fisher Scientific, 4346907) sealed with adhesive film. Reactions were prepared with 10 μL of Fast SYBR^™^ Green Master Mix (Thermo Fisher), 7 μL of nuclease-free water, 1 μL of each of the forward and reverse primers prepared to a stock concentration of 10 μM, and 1 μL of cDNA. Reactions for each biological replicate were performed with either technical duplicates or triplicates and data averaged to ensure reproducibility. All data are given as fold change relative to housekeeping genes *18S* or *Oaz1*. Primer sequences used are given in Table S3.

### QUANTIFICATION AND STATISTICAL ANALYSIS

#### Semi-automated quantification of clonal parameters

##### Clone size distributions

To extract information on clonal dynamics, the clone size distributions for each sample, which depict the probability of observing clones larger than a given size within the dataset, were constructed using all clones identified in imaged lobe pieces except for singlets. In both *Confetti* and *Red2Kras* samples, these distributions were generated by computing the complementary cumulative distribution function separately for each mouse at a given timepoint to appropriately assess mouse-to-mouse variability. The average distribution between mice was also later computed to allow direct comparison of distributions across distinct timepoints in single unified plots for each mouse model. Clones labelled with either YFP or RFP in Confetti mice were first analyzed independently to assess fluorophore-specific differences between the two and later combined for all further analyses following verification of consistent trends in both channels from each sample. Distributions from each mouse across both systems were then fitted with a minimal mathematical model to extract information on the compositions and cycling rates of AT2 stem cell behavior (Methods S1).

##### Clone composition

Clonal composition was deduced by measuring the proportion of cells expressing pro-Sftpc as a proxy for AT2 identity. For every clone, the level of pro-Sftpc expression within individual cells was determined by binarizing the pro-Sftpc channel to count the number of labelled pixels within each clone. Following this, the total number of pro-Sftpc^+^ pixels was normalized to account for the number of cells expressing pro-Sftpc by measuring the typical pro-Sftpc expression in singlets in each sample independently.

##### Clone density

To quantify the density of labelled cells and labelled clones across the tissue, the average cross-sectional area of each sampled lobe piece was first estimated using a 2D projection of the DAPI channel in the tiled z-stack image. Cross-sectional lobe area was then defined using the whole region, including the airways. This parameter was used to normalize the number of labelled clones and labelled cells in that lobe piece to obtain clone density and labelled cell density respectively.

##### YFP-RFP size-distance correlation

To assess proximity-based effects, the size of each YFP^+^ clone was measured as a function of its distance to the nearest RFP+ mutant clone at 50 μm intervals. A similar approach was used to determine the effect of mutant clones on YFP^+^ clonal composition, with the fraction of pro-Sftpc^–^cells in each wildtype clone measured as a function of distance from the nearest RFP^+^ clone. Since numerous clones were induced in each mouse, the number of YFP^+^ clones decreased with increasing distance from RFP^+^ clones, limiting the statistical analysis to a resolution of 300–400 μm.

#### Manual image quantification

##### Analyses of tissue sections

All manual image processing and analyses, excepting representative 3D clonal reconstructions, were performed in Fiji (ImageJ)^49^ using the CellCounter plugin. Where the number of DAPI^+^ nuclei needed to be quantified, images were binarized by manually adjusting the detection threshold. Overlapping nuclei were separated using the watershed function and individual nuclei were automatically detected by particle analysis using a minimum particle size of 5 μm.

##### Analyses of organoid images

Organoid formation efficiencies and organoid sizes were quantified on day 14 of culture in a semi-automated fashion using images obtained from each live well, which were binarized in Fiji and where necessary, the watershed function was implemented to separate fused organoids. Both the number and 2D cross-sectional area of all organoids within each well were detected through particle analysis, with particles larger than 1000 μm^2^ considered as organoids. Formation efficiencies were calculated based on the total number of organoids detected versus the number of cells initially plated per well (5,000–10,000 cells in all cases). For doxycycline-treated cultures, only ZsGreen^+^ organoids were quantified, while all mutant RFP^+^ ZsGreen^–^organoids were quantified in control (-Dox) wells. All IF stained organoid images were analyzed and processed in Fiji.

#### Statistical analyses

A minimum of *n* = 3 biological replicates or independent experiments were used for all statistical analyses, with the exact number of replicates provided in the figure legends. All confocal images and other analyses are representative of a minimum of *n* = 3 biological replicates, unless explicitly stated otherwise. Total numbers of clones analyzed per sample for mathematical modelling are provided in Table S1.

All data are presented as either mean ± standard deviation (SD) or mean ± standard error of the mean (SEM) as specified. All graphs were prepared using either MATLAB, GraphPad Prism 8 or R. Statistical comparisons between cumulative clone size distributions were computed using Kolmogorov-Smirnov tests. In all other cases, statistical significance was determined between groups using the one-way Analysis of Variance (ANOVA) with a Tukey’s or Dunnett’s multiple comparisons test as appropriate, or via two-tailed unpaired or paired Student’s t-tests, as specified in the figure legends.

## Supplementary Material

Methods S1

Supplemental Figures

**Document S1.** Figures S1–S7 and Tables S1–S3.

**Methods S1.** The minimal two-compartment model. Related to STAR Methods.

## Figures and Tables

**Table:** Key resources table

REAGENT or RESOURCE	SOURCE	IDENTIFIER
Antibodies		
Rat anti-Ager	R&D Systems	Cat# MAB1179; RRID:AB_2289349
Rabbit anti-caveolin-1 (D46G3)	Cell Signaling Technology	Cat# 3267; RRID:AB_2275453
Rabbit anti-CD177	R&D Systems	Cat# MAB8186; RRID:AB_3659071
Rabbit anti-cleaved caspase-3 (Asp175)	Cell Signaling Technology	Cat# 9661; RRID:AB_2341188
Rat anti-CD49b (Itga2) (DX5)	Thermo Fisher Scientific	Cat# 14-9571-85
Rabbit anti-Itga2 (EPR17338)	Abcam	Cat# ab181548; RRID:AB_2847852
Rat anti-Ki67 (SolA15)	Thermo Fisher Scientific	Cat#14-5698-82; RRID:AB_10854564
Rat anti-cytokeratin 8 (Krt8)	DSHB	Cat# TROMA-I; RRID:AB_531826
Rat anti-DC-LAMP (Lamp3 / CD208)	Dendritics	Cat# DDX0191P-100; RRID:AB_2827532
Rabbit anti-Lpcat1	Proteintech	Cat# 16112-1-AP; RRID:AB_2135554
Hamster anti-T1a (Pdpn)	DSHB	Cat# 8.1.1; RRID:AB_531893
Rabbit anti-phospho-ERK	Cell Signaling Technology	Cat# 4370; RRID:AB_2315112
Rabbit anti-prosurfactant protein C	Millipore	Cat# AB3786; RRID:AB_91588
Rabbit anti-RasG12D mutant (D8H7)	Cell Signaling Technology	Cat# 14429, RRID:AB_2728748
Goat anti-CC10 (Scgb1a1) (T-18)	Santa Cruz Biotechnology	Cat# sc-9772; RRID:AB_2238819
Goat anti-SPC (Sftpc)	Santa Cruz Biotechnology	Cat# sc-7706; RRID:AB_2185507
Rabbit anti-Sox9 (EPR14335)	Abcam	Cat# ab185230; RRID:AB_2715497
Goat anti-tdTomato	SICGEN	Cat# AB8181; RRID:AB_2722750
Alexa Fluor^®^ 647 donkey anti-Goat IgG (H+L)	Thermo Fisher Scientific	Cat# A-21447; RRID:AB_2535864
Alexa Fluor^®^ 647 goat anti-Syrian Hamster IgG (H+L)	Thermo Fisher Scientific	Cat# A-21451; RRID:AB_2535868
Alexa Fluor^®^ 647 donkey anti-Rabbit IgG (H+L)	Thermo Fisher Scientific	Cat# A-31573; RRID:AB_2536183
Alexa Fluor^®^ 488 donkey anti-Rat IgG (H+L)	Thermo Fisher Scientific	Cat# A-21208; RRID:AB_2535794
DyLight 755 donkey anti-Goat IgG (H+L)	Thermo Fisher Scientific	Cat# SA5-10091; RRID:AB_2556671
DyLight 755 donkey anti-Rabbit IgG (H+L)	Thermo Fisher Scientific	Cat# SA5-10043; RRID:AB_2556623
DyLight 755 donkey anti-Rat IgG (H+L)	Thermo Fisher Scientific	Cat# SA5-10031; RRID:AB_2556611
PE/Cyanine7 anti-mouse CD326 (Ep-CAM)	BioLegend	Cat# 118216; RRID:AB_1236471
Alexa Fluor^®^ 647 rat anti-mouse CD177	BD Biosciences	Cat# 566599; RRID:AB_2869790
Pacific Blue^™^ anti-mouse CD49b (Itga2)	Biolegend	Cat# 108918; RRID:AB_2265144
APC Rat Anti-Mouse CD45	BD Biosciences	Cat# 559864; RRID:AB_398672
APC Rat Anti-Mouse CD31 (MEC13.3)	BD Biosciences	Cat# 551262; RRID:AB_398497
FITC MHC Class II (I-A/I-E) monoclonal antibody (M5/114.15.2)	eBioscience	Cat# 11-5321-81; RRID:AB_465231
Bacterial and virus strains		
pLVX-EF1a-Tet3G	Clontech	Cat# 631359
pLVX-TRE3G-ZsGreen	Clontech	Cat#631164
		
		
Biological samples		
		
		
Chemicals, peptides, and recombinant proteins		
Tamoxifen	Sigma	Cat# T5648
Corn oil	Sigma	Cat# C8267
EdU (5-ethynyl-2’-deoxyuridine)	Invitrogen	Cat# A10044
Normal Donkey Serum	Jackson ImmunoResearch	Cat# 017-000-121
DAPI	Sigma	Cat# D9542
RapiClear 1.52	SunJin Lab	Cat# RC152002
Dispase (50U/mL)	Corning	Cat# 354235
Collagenase/dispase solution	Sigma	Cat# 10269638001
DNase I	Sigma	Cat# D4527
Gibco HEPES (1M)	Invitrogen	Cat# 15630080
BMS-345541	Abcam	Cat# ab144822
Recombinant murine IL-1β	Peprotech	Cat# 211-11B
Recombinant mouse Dlk-1	R&D Systems	Cat# 8545-PR-050
Recombinant mouse osteopontin (Spp1)	R&D Systems	Cat# 441-OP-050
TransIT^®^-LT1 Transfection Reagent – MIR 2300	GeneFlow	Cat# E7-0002
ROCK inhibitor Y-27632	Cambridge Bioscience	Cat# SM02-1
Certified Low Melt Agarose	Bio-Rad	Cat# 1613111
TRIzol^™^ Reagent	Invitrogen	Cat# 15596026
Corning^®^ Matrigel^®^ Growth Factor Reduced (GFR) Basement Membrane Matrix	Corning	Cat# 356231
B-27^™^ Supplement (50X), serum free	Gibco	Cat# 17504044
Recombinant FGF7	Peprotech	Cat# 100-19-100
FGF10	Peprotech	Cat# 100-26-100
Noggin	Peprotech	Cat# 250-38
Recombinant EGF	Life Technologies	Cat# PMG8043
Chiron (CHIR99021)	Tocris Bioscience	Cat# 4423
Amphotericin B	Sigma	Cat# A2942
DNase I, Amplification Grade	Invitrogen	Cat# 18068015
Fast SYBR^™^ Green Master Mix	Thermo Fisher Scientific	Cat# 4385616
Doxycycline Hyclate	Sigma	Cat# D5207
Critical commercial assays		
In-Fusion HD cloning kit	Clontech	Cat# 638951
Alexa Fluor^™^ 647 Click-iT^™^ Plus EdU Cell Proliferation Kit	Invitrogen	Cat# C10640
SuperScript^™^ IV First-Strand Synthesis System	Thermo Fisher Scientific	Cat# 18091050
Deposited data		
scRNA-sequencing of *in vivo* AT2 cells during homeostasis and oncogenesis	This paper	GSE247505
scRNA-sequencing for *in vivo* AT2-lineage during regeneration	Choi et al.^11^	GSE145031
Mendeley Data Figure 1	This paper	https://doi.org/10.17632/ss6pb96pty.1
Code to extract clonal properties and perform statistical modeling-based analyses	This paper	https://doi.org/10.5281/zenodo.14625398
		
Experimental models: Cell lines		
		
		
Experimental models: Organisms/strains		
Mouse: *Sftpc-CreERT2*: B6.129S-Sftpc^tm1(cre/ERT2)Blh^/J	Barkauskas et al.^47^; The Jackson Laboratory	RRID:IMSR_JAX:028054
Mouse: *R26R-Confetti*: Gt(ROSA)26Sor^tm1(CAG-Brainbow2.1)Cle^/J	Snippert et al.^33^; The Jackson Laboratory	RRID: IMSR_JAX:013731
Mouse: *Il1r1-CreERT2*: Il1r1^tm1(cre/ERT2)Jhle^	Choi et al.^11^	MGI:6718882
Mouse: *R26R-ZsGreen*: B6.Cg-*Gt(ROSA)26Sor*^*tm6(CAG-ZsGreen1)Hze*^/J	The Jackson Laboratory	RRID:IMSR_JAX:007906
Mouse: *Red2KrasG12D*: Gt(ROSA)26Sor^em2(CAG-Brainbow2.1,-Kras*)Koo^	Yum et al.^25^	MGI:7259836
Mouse: *Il1r1*^*loxP/loxP*^: B6.129(Cg)-*Il1r1*^*tm1.1Rbl*^/J	Robson et al.^48^; The Jackson Laboratory	RRID:IMSR_JAX:028398
Mouse: NSG: NOD.Cg-*Prkdc*^*scid*^ *Il2rg*^*tm1Wjl*^/SzJ	Ishikawa et al.^52^;The Jackson Laboratory	RRID:IMSR_JAX:005557
Oligonucleotides		
Primers for qPCR, see Table S3	This paper	N/A
		
Recombinant DNA		
		
Software and algorithms		
Leica LAS X	Leica	https://www.leica-microsystems.com/products/microscope-software/p/leica-las-x-ls/
Imaris x64 (v9.4.10)	Oxford Instruments	https://imaris.oxinst.com
Fiji (ImageJ)	Schindelin et al.^53^	https://imagej.net/software/fiji/index
MATLAB R2020a	MathWorks	https://www.mathworks.com/products/matlab.html
CellRanger (v3.1.0)	10x Genomics	https://www.10xgenomics.com/support/software/cell-ranger/
Seurat (v4.3.0)	Hao et al.^49^	https://satijalab.org/seurat/
R (v4.2.1)	N/A	
ComplexHeatmap (v.2.15.1)	Gu et al.^50^	https://bioconductor.org/packages/release/bioc/html/ComplexHeatmap.html
CellChat (v1)	Jin et al.^29^	http://www.cellchat.org
FlowJo (v10.9.0)	BD	https://www.flowjo.com
GraphPad Prism 8	GraphPad	https://www.graphpad.com/scientific-software/prism/www.graphpad.com/scientific-software/prism/
Other		
μ-Slide 8 Well	ibidi	Cat# 80826
Nunc^™^ Lab-Tek^™^ 8-well chamber slides	Thermo Fisher Scientific	Cat# 154534
		
