# Supplementary material for: Sustained NF-κB activation allows mutant alveolar stem cells to co-opt a regeneration program for tumor initiation
Source: Cell Stem Cell. Author manuscript; Available in PMC 2026 Aug 3. (PMC13432921; doi:10.1016/j.stem.2025.01.011)
Supplement: Methods S1 [file NIHMS2194192-supplement-Methods_S1.pdf]

Methods S1: The minimal two-compartment model. Related to STAR Methods

Here we provide details on the minimal two-population model proposed to describe the clonal dynamics. First, we discuss how the experimental observations provide evidence for the existence of two distinct cell populations, a slower and a faster cycling one. To characterise these populations, we introduce a minimal model for the clone size distribution, which allows us to mathematically infer the clone size threshold at which the distribution becomes dominated by the faster-cycling population. After introducing the model and discussing its predictions, we describe the protocol to test and fit the model to the experimental data, and provide tables with the fitted parameters.

Two-population model

To extract information on the clonal dynamic, we constructed the clone size distributions for each sample (lung), combining the clones identified in each lobe and focusing only on the proliferative population, i.e., clones with two or more cells. We note that in the control (confetti) samples, the YFP+ and RFP+ channels showed consistent trends in the clone size distributions, which allowed us to combine the information of both channels for these mice.

Specifically, we analysed the cumulative distribution, which measures the probability of observing a clone larger than a given size. From this, we noticed that the clones had a large dispersion characterised by a long tail decay, where the cumulative distribution was well fitted by a bi-exponential decay. In a log-linear plot, this behaviour is evidenced by two distinct decays: one characterising the small clone behaviour and another characterising the

large clone decay. As discussed in the main text, this bi-exponential behaviour is likely to be caused by two distinct independent cell populations, with different proliferative capacities (i.e. different expansion rates, defined below as $\Delta_{F,S}$, with $\Delta_F > \Delta_S$): A fast cycling population will contribute to form the tail of the distribution, dominated by large clones, while slow cycling cells contribute primarily to the small clone decay.

In this picture, we considered a minimal two population model consisting of a fast cycling population (F) and an independent, slow cycling population (S), where cells in each compartment choose stochastically (probabilistically) between cell division and loss.

$$
\begin{aligned}
F &\xrightarrow{\sigma_F} \begin{cases} F+F & \text{Pr. } r_F \\ 0 & \text{Pr. } 1-r_F \end{cases} \\
S &\xrightarrow{\sigma_S} \begin{cases} S+S & \text{Pr. } r_S \\ 0 & \text{Pr. } 1-r_S \end{cases}
\end{aligned}
\tag{1}
$$

where F and S cell cycle at rates σ_F and σ_S, respectively. We note here that, biologically, cell loss may occur either through cell differentiation and loss via symmetric or asymmetric division, or via direct cell death. However, as long as the intermediate, differentiated state is short-lived, the minimal model presented here will exhibit the same dynamics as one where the intermediate state is considered explicitly. In the context of a duplication and loss dynamic, fast and slow clone expansions occur with effective expansion rates $\Delta_F = \sigma_F(2r_F - 1)$ and $\Delta_S = \sigma_S(2r_S - 1)$. As the dynamics of each sub-population correspond to a loss and renewal process, their individual distributions of clone sizes follow an exponential decay (see for instance [1]). Now, because the two compartments, F and S, are considered independent, i.e., there is no differentiation of cells from one compartment into cells of the other, the probability $T_n(t)$ of finding a clone of n cells, a time t after induction of a single cell, is given by the sum of two exponential distributions, referred to here as a bi-exponential distribution

$$T_n(t) = f_S n_S(t) \mathrm{e}^{-n/n_S(t)} + (1 - f_S) n_F(t) \mathrm{e}^{-n/n_F(t)}, \tag{2}$$

where $n_S(t)$ and $n_F(t)$ correspond to the average clone sizes of the S (slow) and F (fast) populations, respectively, and f_S accounts for the fraction of induced cells that belong to the S population. In the homeostatic regime (where the probabilities of renewal and loss are balanced), we expect the loss and renewal dynamic to be driven by neutral competition

between clones, leading to a temporal decline in clone number and a linear increase in the average clone size (proportional to the effective duplication rate i.e. $n(t) \propto r\sigma t$), as is clearly seen in the confetti data in the 1 to 72 week range. However, upon oncogene activation, expansion dominates over loss ($r_S > 1/2$ and $r_F > 1/2$) and the average clone sizes grow exponentially with time: $n_S(t) \propto \mathrm{e}^{\Delta_S t}$ and $n_F(t) \propto \mathrm{e}^{\Delta_F t}$, where the $\Delta_S \ll \Delta_F$ at all times.

As long as $0 < \Delta_S \ll \Delta_F$, the model predicts a biexponential distribution for the clone size distribution, with F-derived clones duplicating in size at a much faster rate than S-derived ones. By fitting the model to the data, we can then infer mathematically the characteristic clone size at which the distribution becomes dominated by faster-cycling (larger) clones. To fit this biexponential distribution of clone sizes to the data, we focused exclusively on the proliferative population ($n > 1$) and used the cumulative probability of clone sizes to fit the theoretical expression (as was done, for example, in [2])

$$C_{n>1}(t) = 1 - \sum_{m=2}^{n} T_{m>1}(t) = \frac{1-f_S}{1-s(t)}\mathrm{e}^{-n/n_F(t)} + \frac{f_S}{1-s(t)}\mathrm{e}^{-n/n_S(t)}, \quad (3)$$

where $s(t) = T_0 + T_1$ ensures normalisation. By fitting our model to the data, we could extract three key parameters: the average clone sizes of the large $n_F(t)$ and small $n_S(t)$ clones as a function of time, and the fraction of induced slow cycling cells f_S of the expanding population, see the Fitting strategy section.

Fitting strategy

The parameters in the model: σ_F, r_F, σ_S, r_S and f_S, can be constrained significantly by the experimental observations. From the average clone size over time, we may extract estimated parameters for the duplication rate (for the balanced case, where $r_{F,S} = 1/2$, or expansion, where $r_{F,S} > 1/2$). For this, we fit the theoretical prediction of the cumulative distribution of clone sizes, Eq. (3), to the cumulative distribution obtained from the clonal labelling experiments, for each sample and at every time-point separately. From this, we could extract estimated parameters and corresponding confidence intervals for the fraction of induced fast cycling cells f_F and average clone sizes over time, $\bar{n}_F$ and $\bar{n}_S$ (see “biexp fits” in Table 1), which we used as inputs in our numerical simulations of the full stochastic model to find best fit parameters, as discussed below.

Fitting the bi-exponential decay to the cumulative distribution of clone sizes allowed us to

find the typical clone size where the distribution transition threshold to the long term decay. With this threshold, we could then segregate the clone sizes into smaller (slower cycling) and large (faster cycling) clones.

Confetti

In the case of the control (confetti) samples, we observed a linear growth of the average clone size as a function of time. This was true either when analysing the whole pool of clones or when segregating according to slow and fast clones. This linear behaviour is consistent with a loss and replacement dynamic, where the two processes are balanced and thus, occur with equal probabilities. This observation fixed the duplication probabilities of both populations to $r_F = r_S = 1/2$. Moreover, in such a balanced process, the growth in the average clone size is proportional to the duplication rate, which in our setup are equal to $r_F\sigma_F$, for the fast, and $r_S\sigma_S$ for the slow populations. Considering the estimated parameters and confidence intervals found from the bi-exponential fits to the individual time-points, we then performed numerical simulations of the full stochastic model, to find best fit parameters. For this, we performed simultaneous fits of the model to the 12 to 72 weeks post-induction data.

The best fit parameters found through this procedure can be seen in the "Best fit" column of Tables 1.

Kras mutant: YFP$^+$ (WT) and Kras$^+$ clones

Similarly to the control, WT clones in the Kras-induced samples also exhibited a linear-like increase in their size over time, consistent with a balanced output of duplication and loss. However, in this case, the average clone sizes appeared to expand much more rapidly in the first week post-induction compared to the expansion from 1 to 4 weeks post induction. To properly account for this "two-stage" behaviour in the numerical simulations, we fitted the two regimes independently, first focusing only on the first, fast growth stage, which provided an estimate for the fraction of induced fast cycling cells and duplication rates in the initial growth regime, σ_S and σ_F, for the slow and fast populations, respectively. After finding the best fits to the initial growth, we found best fits to the second, slower growth stage, given by cycling rates σ'_S and σ'_F, for the slow and fast populations, respectively. It should be noted that in both stages, the slower cycling population exhibited cycling rates much smaller than

those of the faster cycling population.

For the Kras$^+$ clones, the behaviour of the average clone sizes over time exhibited a clear exponential growth rate, consistent with a loss of fate balance, biased towards duplication. Here, we found a slowdown in the expansion dynamic, likely due to saturation of growth at very large clone sizes. This meant that up to the 2 weeks time-point, the average size followed an exponential growth with fixed expansion rate, Δ_S and Δ_F, which decreased significantly from 2 to 4 weeks post-induction, to constant values Δ'_S and Δ'_F. For the Kras$^+$ clones, we considered the fraction of induced fast cycling cells found for the WT fits. The exponential growth of the average clone sizes indicated an imbalance in the cell division output, biased towards cell duplication, here we fixed the duplication probabilities of both populations to $r_F = r_S = 0.7$.

The best fit parameters for the WT and mutant clones are shown in the Best fit column of Tables 2 and 3, respectively.

Stochastic simulations of the two-population model

The average behaviour of the two-population model is well described by the clone size distribution (2). However, to validate our model and to capture the variability in the data that originates from the stochastic (probabilistic) nature of the cell cycling and decision-making dynamics, we turned to stochastic simulations. For this, we implemented a standard Gillespie algorithm [3] in order to simulate the evolution of fast and slow clones. In this algorithm, we simulate the evolution for the number of cells in a clone. Initialising a clone with a single cell, $N(t_0) = 1$, at time $t = t_0 = 0$, which cycles at rate σ, duplicates with probability r and is lost with probability $1 - r$, the number of cells at time $t = t_0 + \Delta t$ is computed as follows:

1. First, we compute the propensity function $w = w_{\text{div}} + w_{\text{loss}}$, where $w_{\text{div}} = \sigma r N(t)$, $w_{\text{loss}} = \sigma(1 - r)N(t)$, and define the effective division and loss rates.
2. Then, we update the time to $t + \Delta t$, where $\Delta t = -\frac{1}{w} \ln r_1$, $r_1 \in (0, 1]$ uniformly distributed random number.
3. Finally, we choose a random cell in the clones, and decide whether it duplicates or dies

in the time interval Δt according to

$$N(t+\Delta t) = \begin{cases} N(t)+1 & r_2 \leq w_{div}/w \\ N(t)-1 & \text{otherwise}, \end{cases}$$

where $r_2 \in (0,1]$ is a uniformly distributed random number, different from r_1.

4. Steps (1)-(3) are repeated until the maximum simulation time $t = t_{\max}$ is reached. Here $t_{\max}$ corresponds to the time after confetti or Kras induction, in each case.

As a single run of the algorithm described here simulates the temporal evolution of the number of cells in a single clone, we must run a large number of repeats to construct the distributions of clone sizes. Here, we simulated a total number of 10^3 realisations, each for 10^3 clones. Of the 10^3 clones in each realisation of the model, we simulated a fraction $f_F = 1 - f_S$ of the clones with parameters corresponding to the fast cycling populations (σ_F and r_F) and a fraction f_S of the clones with parameters corresponding to the slow cycling populations (σ_S and r_S). From this, we could construct the distribution of clone sizes for the model, with mean and SD, as shown in the plots for the cumulative distribution of clone sizes. The results from stochastic simulations were compared to the experimental data as discussed in the Goodness of fit section.

Goodness of fit

To assess the goodness of fit of the stochastic simulations to the experimental data, we focused on the cumulative distribution of clone sizes and measured the root-mean-square-log-error (RMSLE) between the two, defined as

$$RMSLE = \sqrt{\frac{1}{n}\sum_{m=2}^{n}(\log C_m^{\text{th}} - \log C_m^{\text{exp}})^2}. \quad (4)$$

Here C_m^{th} and C_m^{exp} are the theoretical and experimental cumulative distributions, respectively, and n is the maximum clone size for which both cumulative distributions are non-zero.

The logarithm of the cumulative distribution is taken to properly account for the exponential nonlinear decay in the data, providing equal weights to the head and tail of the distributions. A value of the RMSLE closer to zero corresponds to a better fit.

For the values of the RMSLE obtained from the best fits of the model to the experimental data, see Table 4.

Tables of parameters

Confetti

Parameter	biexp fit (95% C.I.)	best fit
f_S	0.1 (0.0,0.2)	0.16
σ_S (1/week)	0.021 (0.0069, 0.036)	0.03
σ_F (1/week)	0.124 (0.035, 0.21)	0.124

TABLE 1: Parameters considered in the numerical simulations of the stochastic two-population model for the confetti data. The second column (biexp) shows the estimated parameters from the biexponential fit of Eq. (3), to the experimental cumulative distributions of clone sizes. The third column (best fit), shows the best fit parameters found through numerical simulations of the full stochastic two-population model.

YFP$^+$ (Kras)

Parameter	biexp fit (95% C.I.)	best fit
f_S	0.06 (0.0,0.13)	0.16
σ_S (1/week)	2.136	1.5
σ_F (1/week)	13.62	13.62
σ'_S (1/week)	0.2425 (0.0, 0.58)	0.2425
σ'_F (1/week)	1.221 (0.0, 3.0)	1.221

TABLE 2: Parameters considered in the numerical simulations of the stochastic two-population model for the YFP$^+$ (WT) clones of the Kras data. Here, we consider that the result of cell divisions is balanced, i.e., $r_F = r_S = 1/2$. The second column (biexp) shows the estimated parameters from the biexponential fit of Eq. (3), to the experimental cumulative distributions of clone sizes. The third column (best fit), shows the best fit parameters found through numerical simulations of the full stochastic two-population model.

$Kras^+$ (Kras)

Parameter	biexp fit (95% C.I.)	best fit
f_S	-	0.16
Δ_S (1/week)	0.9 (0.72, 2.6)	1.1
Δ_F (1/week)	2.9 (1.6, 4.3)	3.1
Δ'_S (1/week)	0.36	0.01
Δ'_F (1/week)	0.96	0.5

TABLE 3: Parameters considered in the numerical simulations of the stochastic two-population model for the RFP^+ ($Kras^+$) clones of the Kras data. Here, we consider that the result of cell divisions is biased towards duplication, with a choice of $r_F = r_S = 0.7$. The second column (biexp) shows the estimated parameters from the biexponential fit of Eq. (3), to the experimental cumulative distributions of clone sizes. The third column (best fit), shows the best fit parameters found through numerical simulations of the full stochastic two-population model.

Goodness of fit

Confetti	12 weeks	24 weeks	36 weeks	52 weeks	60 weeks	72 weeks
RMSLE	0.46	0.76	0.55	0.61	0.29	0.53

YFP^+ (Kras)	1 week	2 weeks	4 weeks
RMSLE	3.09	0.40	0.49

RFP^+ (Kras)	1 week	2 weeks	4 weeks
RMSLE	4.57	0.32	3.91

TABLE 4: Root-mean-square-log-error (RMSLE) of the best fit of the theory to the experimental cumulative distributions of clone sizes.

References

[1] AM Klein and BD Simons. Universal patterns of stem cell fate in cycling adult tissues. *Development*, 138(15):3103–3111, 2011.

[2] P Karras et al. A cellular hierarchy in melanoma uncouples growth and metastasis. *Nature*, 610(7930):190–198, 2022.

[3] R Erban and SJ Chapman. *Stochastic modelling of reaction–diffusion processes*, volume 60. Cambridge University Press, 2020.
