# Supplementary material for: Sustained NF-κB activation allows mutant alveolar stem cells to co-opt a regeneration program for tumor initiation
Source: Cell Stem Cell. Author manuscript; Available in PMC 2026 Aug 3. (PMC13432921; doi:10.1016/j.stem.2025.01.011)
Supplement: Supplemental Figures [file NIHMS2194192-supplement-Supplemental_Figures.pdf]

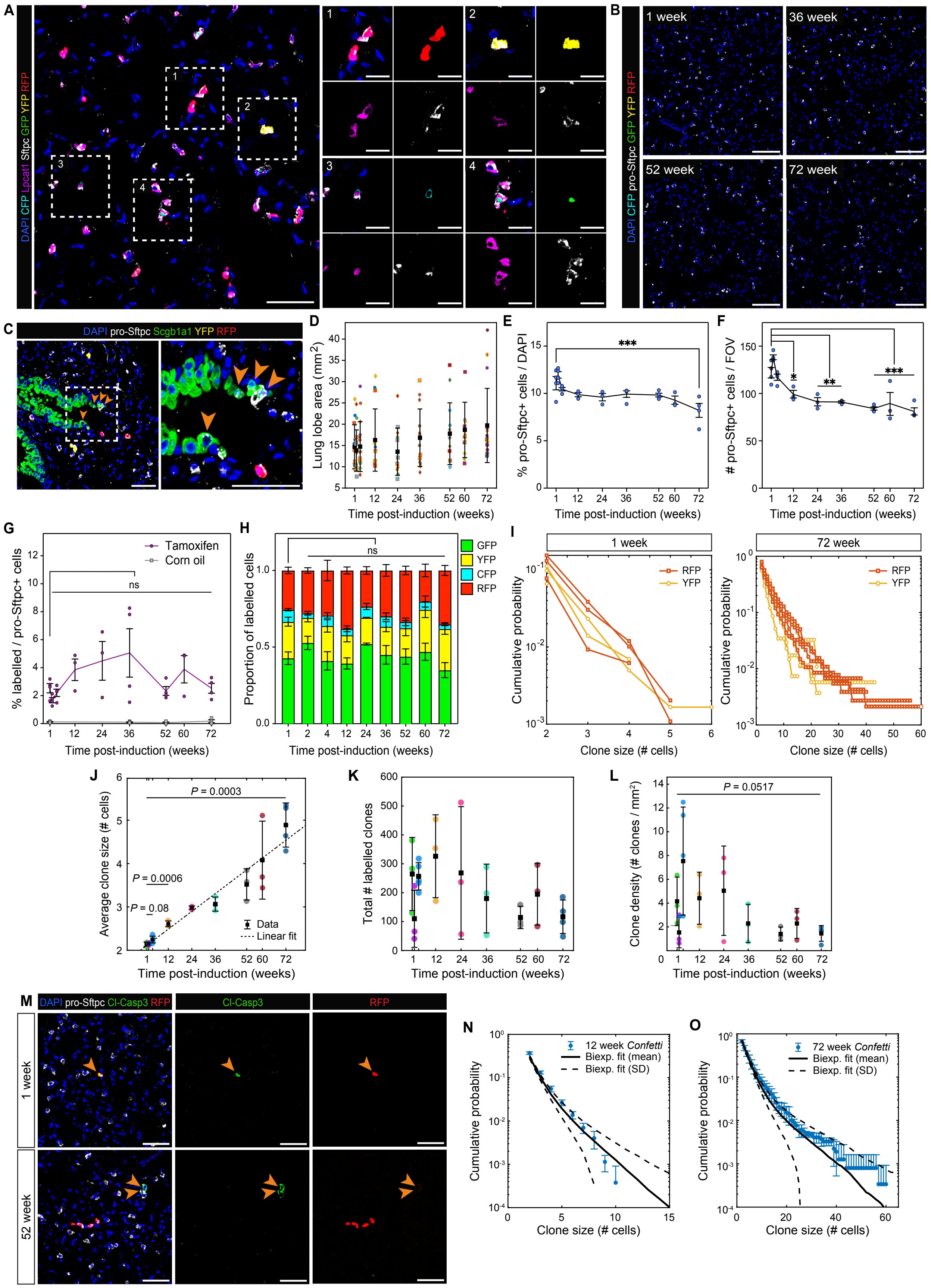

Figure S1. Homeostatic AT2 clones display long-term heterogeneity that cannot be explained by cell loss through apoptosis. Related to Figure 1.

(**A**) Representative confocal images of *Confetti* lungs administered with 3 x 0.2 mg/gbw tamoxifen at 1-week post-induction, showing expression of key AT2 markers Sftpc (white) and Lpcat1 (magenta). Right panels are magnifications of left panel. Scale bars, 50 μm (left) and 20 μm (right). DAPI (blue), GFP (green), YFP (yellow), RFP (red), and CFP (cyan).

(**B**) Representative confocal images of 1-week, 36-week, 52-week, and 72-week *Confetti* lungs administered with corn oil, showing no leaky recombination events. DAPI (blue), pro-Sftpc (white), GFP (green), YFP (yellow), RFP (red), and CFP (cyan). Scale bars, 100 μm.
(**C**) Representative confocal images of the bronchioalveolar duct junction in 1-week *Confetti* lungs showing lineage-labelled cells and bronchioalveolar stem cells (BASCs) are mutually exclusive. Right panel, magnifications of left panel. Orange arrowheads, dual Scgb1a1$^+$ pro-Sftpc$^+$ putative BASCs. DAPI (blue), pro-Sftpc (white), Scgb1a1 (green), YFP (yellow), and RFP (red). Scale bars, 50 μm.
(**D**) 2D cross-sectional area of individual lung lobe pieces sampled throughout the lineage tracing period. Each dot represents 1 lobe with a minimum of 4 lobes quantified per mouse. Data are represented as mean ± SD.
(**E**) Percentage of pro-Sftpc$^+$ AT2 cells relative to DAPI$^+$ nuclei.
(**F**) Absolute number of pro-Sftpc$^+$ AT2 cells detected per field of view (FOV) under a 20x objective.
For (E) and (F), each dot represents 1 mouse. Data are represented as mean ± SEM. *$p < 0.05$; **$p < 0.01$; ***$p < 0.001$ relative to the 1-week timepoint, one-way ANOVA with Dunnett's multiple comparisons test.
(**G**) Percentage of lineage-labelled cells relative to all pro-Sftpc$^+$ cells over time. Equivalent corn oil quantifications shown in grey. Each dot represents 1 mouse. $n = 3$ mice for corn oil-treated cohorts.
(**H**) Proportion of lineage-labelled cells expressing each fluorescent reporter per timepoint.
For (G) and (H), data are represented as mean ± SEM. ns, not significant relative to the 1-week timepoint, one-way ANOVA with Dunnett's multiple comparisons test.
(**I**) Cumulative size distributions of 1-week (left) and 72-week (right) YFP$^+$ and RFP$^+$ clones, showing similar distributions in both compartments. Each line represents one mouse.
(**J**) Average homeostatic clone sizes over time. Each dot represents 1 mouse. Data are represented as mean ± SD. *p* values given in the panel, unpaired Student's t tests.
(**K**) Total number of labelled clones (excluding singlets) quantified over time.
(**L**) Clone density across the tissue over time with total number of clones normalized against lobe area in mm^2. All values were not significant relative to the 1-week timepoint, with the *p* value at 72 weeks given in the panel, unpaired Student's t-tests.
For (K) and (L), each dot represents 1 mouse. Data are represented as mean ± SD.
(**M**) Confocal images demonstrating rare Cl-Casp3 (green) expression in both labelled and unlabeled AT2 cells. DAPI (blue), pro-Sftpc (white), and RFP (red). Scale bars, 50 μm.
(**N**), (**O**), Cumulative size distributions of 12-week (N) and 72-week (O) clones. Black line, biexponential fit; dashed line, SD of fit.
For (D)–(L), (N), and (O), $n \geq 3$ mice per timepoint (total numbers of clones and mice analyzed per timepoint are given in Table S1).
All images representative of $n = 3$ mice.

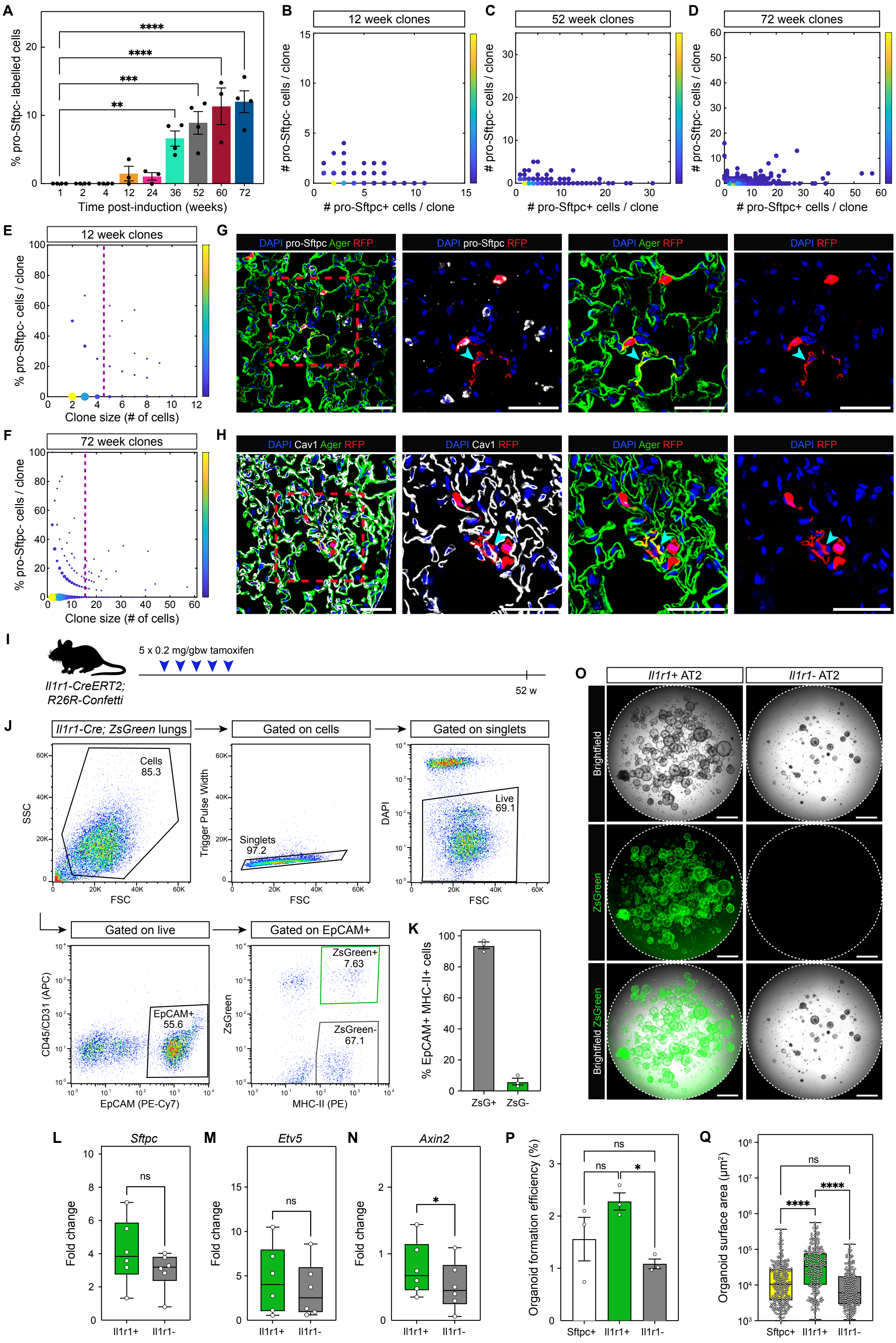

A
% pro-Sftpc- labelled cells
Time post-induction (weeks)
B
12 week clones
C
52 week clones
D
72 week clones
pro-Sftpc- cells / clone
pro-Sftpc+ cells / clone
E
12 week clones
F
72 week clones
% pro-Sftpc- cells / clone
Clone size (# of cells)
G
DAPI pro-Sftpc Ager RFP
DAPI pro-Sftpc RFP
DAPI Ager RFP
DAPI RFP
H
DAPI Cav1 Ager RFP
DAPI Cav1 RFP
DAPI Ager RFP
DAPI RFP
I
5 x 0.2 mg/gbw tamoxifen
Il1r1-CreERT2; R26R-Confetti
52 w
J
Il1r1-Cre; ZsGreen lungs
Gated on cells
Gated on singlets
Cells 85.3
Singlets 97.2
Live 69.1
SSC
Trigger Pulse Width
DAPI
FSC
Gated on live
Gated on EpCAM+
EpCAM+ 55.6
ZsGreen+ 7.63
ZsGreen- 67.1
CD45/CD31 (APC)
EpCAM (PE-Cy7)
ZsGreen
MHC-II (PE)
K
% EpCAM+ MHC-II+ cells
ZsG+
ZsG-
O
Il1r1+ AT2
Il1r1- AT2
Brightfield
ZsGreen
Brightfield ZsGreen
L
Sftpc
M
Etv5
N
Axin2
Fold change
Il1r1+
Il1r1-
ns
*
P
Organoid formation efficiency (%)
Sftpc+
Il1r1+
Il1r1-
Q
Organoid surface area (μm²)
****

Figure S2. Slower-cycling and faster-cycling homeostatic AT2 subpopulations maintain long-term self-renewal and differentiation capacity, with faster-cycling cells resembling *Il1r1*⁺ AT2 cells. Related to Figure 1.

(**A**) Percentage of pro-Sftpc⁻ lineage-labelled *Confetti* cells. Each dot represents 1 mouse. Data are represented as mean ± SEM. $^{**}p < 0.01$; $^{***}p < 0.001$; $^{****}p < 0.0001$ relative to the 1-week timepoint, one-way ANOVA with Dunnett's multiple comparisons test.

(**B**), (**C**), (**D**) Number of pro-Sftpc⁻ cells per clone plotted against the number of pro-Sftpc⁺ cells per clone at 12 (B), 52 (C), and 72 (D) weeks post-induction in *Confetti* mice. Color of each dot is scaled according to clone density in that region of the plot.

(**E**), (**F**) Percentage of pro-Sftpc⁻ cells per clone plotted against clone size at 12 (E) and 72 (F) weeks post-induction. Color of each dot is scaled according to clone density in that region of the plot. Dashed line, boundary between slower- and faster-cycling clones.

For (A)–(F), $n \geq 3$ mice per timepoint (total numbers of clones and mice analyzed per timepoint are given in Table S1).

(**G**) Representative confocal images of Ager (green) expression in a small fraction of 72-week homeostatic clones (cyan arrowhead). Ager⁺ cells co-exist in the clone with several pro-Sftpc⁺ (white) lineage-labelled cells as shown.

(**H**) Representative confocal images demonstrating expression of the mature AT1 marker Cav-1 (white) in Ager⁺ (green) RFP⁺ cells (cyan arrowhead) at 72 weeks post-induction.

For (G) and (H), images representative of rare pro-Sftpc⁻ cells observed in YFP⁺ and RFP⁺ clones across $n = 3$ mice. DAPI (blue), pro-Sftpc (white), and RFP (red). Scale bars, 50 μm.

(**I**) Experimental design for lineage tracing homeostatic *Il1r1*⁺ AT2-derived clones. Lungs were collected at the 52-week timepoint for clonal analyses.

(**J**) FACS gating strategy for isolating lineage-labelled *Il1r1*⁺ (ZsGreen⁺) and non-lineage-labelled *Il1r1*⁻ (ZsGreen⁻) AT2 cells from *Il1r1-CreERT2; R26R-ZsGreen* lungs for organoid culture. Samples were gated on cells, singlets, and live before gating on the EpCAM⁺CD45⁻CD31⁻ population to sort ZsGreen⁺ and ZsGreen⁻ MHCII⁺ AT2 cells.

(**K**) Proportion of EpCAM⁺MHCII⁺ AT2 cells in (J) that were *Il1r1*⁻ (ZsGreen⁻) and *Il1r1*⁺ (ZsGreen⁺). Data are represented as mean ± SEM. $n = 3$ mice.

(**L**), (**M**), (**N**) Fold change in mRNA expression levels of *Sftpc* (L), *Etv5* (M), and *Axin2* (N) within *Il1r1*⁺ (ZsGreen⁺) and *Il1r1*⁻ (ZsGreen⁻) AT2 populations. Values are given as fold change relative to bulk AT2 control samples isolated from *Sftpc-CreERT2; R26R-ZsGreen* mice normalized against the housekeeping gene *18S*. Each dot represents 1 independent experiment. Centre line, median; box, interquartile range; whiskers, range. ns, not significant; $^{*}p < 0.05$ relative to *Il1r1*⁻ AT2 cells, paired Student's t-test.

(**O**) Representative fluorescent images of organoids established from *Il1r1*⁺ (ZsGreen⁺) and *Il1r1*⁻ (ZsGreen⁻) AT2 cells. Dotted white circles, edge of the well. Scale bars, 1000 μm.

(**P**) Formation efficiency of organoids in (O). Each dot represents 1 independent experiment.

(**Q**) 2D surface area, in μm^2, of organoids in (P). Each dot represents 1 organoid, with 150 organoids quantified per experiment.

For (P) and (Q), $n = 3$ independent experiments. Centre line, median; box, interquartile range; whiskers, range. ns, not significant; $^{*}p < 0.05$; $^{****}p < 0.0001$, one-way ANOVA with Tukey's multiple comparisons test.

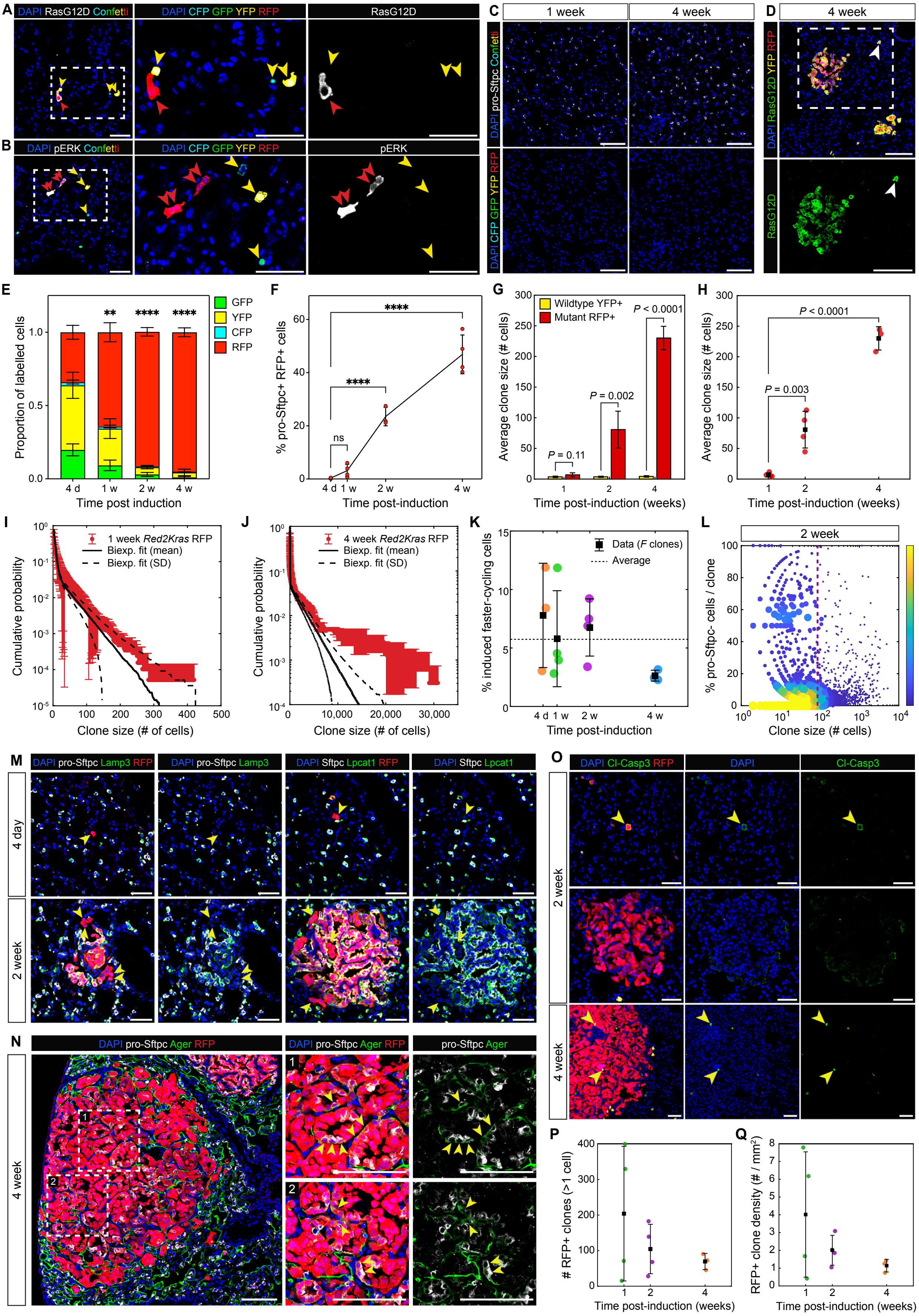

Figure S3. *Red2Kras* mutant clones segregate into slower- and faster-cycling fractions, both of which exhibit evidence of cellular reprogramming. Related to Figure 2.

(**A**), (**B**) Representative confocal images showing RasG12D (white) (A) and phospho-ERK (p-ERK, white) expression (B) localized exclusively to RFP^{+} cells (red arrowheads). Yellow arrowheads, wildtype lineage-labelled

cells negative for RasG12D (A) and pERK (B). DAPI (blue), GFP (green), YFP (yellow), RFP (red), and CFP (cyan). Scale bars, 50 μm.
(**C**) Representative confocal images of 1-week (left) and 4-week (right) *Red2Kras* vehicle control mice, showing no leaky recombination events. DAPI (blue), pro-Sftpc (white), GFP (green), YFP (yellow), RFP (red), and CFP (cyan). Scale bars, 100 μm.
(**D**) Representative confocal images of 4-week *Red2Kras* lungs showing equivalent expression of mutant RasG12D (green) in small (white arrowheads) and large RFP$^+$ clones. DAPI (blue), YFP (yellow) and RFP (red). Scale bars, 100 μm.
(**E**) Proportion of lineage-labelled cells in *Red2Kras* mice expressing each fluorescent reporter per timepoint.
(**F**) Percentage of pro-Sftpc$^+$ mutant cells relative to the total number of pro-Sftpc$^+$ cells in the tissue (both labeled and unlabeled). Each dot represents 1 mouse.
For (E) and (F), data are represented as mean ± SEM. ns, not significant; **$p < 0.01$; ***$p < 0.001$ and ****$p < 0.0001$ relative to the 4-day timepoint, one-way ANOVA with Dunnett's multiple comparisons test.
(**G**) Comparison of average clone sizes in *Red2Kras* mutant RFP$^+$ (red bars) and wildtype YFP$^+$ (yellow bars) compartments.
(**H**) Average mutant RFP$^+$ clone sizes over time. Each dot represents 1 mouse.
For (G) and (H), data are represented as mean ± SD. *p* values are provided in the panels, unpaired Student's t-tests.
(**I**), (**J**) Cumulative size distributions of 1-week (I) and 4-week (J) mutant clones. Black line, biexponential fit; dashed line, SD of fit.
(**K**) Inferred fraction of induced mutant faster-cycling cells obtained from model fits (see Supplementary Note). Dashed line, average value. Data are represented as mean ± SD. Each dot represents 1 mouse.
(**L**) Percentage of pro-Sftpc$^-$ cells per mutant clone plotted against clone size at 2 weeks post-induction. Color and size of each dot is scaled according to clone density at that region of the plot. Dashed line, boundary between slower- and faster-cycling clones.
(**M**) Representative confocal images of loss of pro-Sftpc (white) and Lamp3 (green) expression (left panels) and Sftpc (white) and Lpcat1 (green) expression (right panels) in cells within 4-day (top) and 2-week (bottom) mutant clones. Yellow arrowheads, cells double negative for both markers. DAPI (blue) and RFP (red). Scale bars, 50 μm.
(**N**) Representative confocal images of cells expressing both Ager (green) and pro-Sftpc (white) (yellow arrowheads) in 4-week mutant clones. DAPI (blue), RFP (red), and pro-Sftpc (white). Scale bars, 100 μm.
(**O**) Representative confocal images of elevated Cl-Casp3 (green) expression in small remote RFP$^+$ clones (top), but not larger RFP$^+$ clones (middle), except for rare Cl-Casp3$^+$ cells detected at later timepoints (bottom). DAPI (blue) and RFP (red). Scale bars, 50 μm.
(**P**) Total number of RFP$^+$ clones excluding singlets over time. Each dot represents 1 mouse
(**Q**) Clone density across the tissue over time with total number of clones (excluding singlets) normalized against total lobe area in mm^2. All values were not significant relative to the 1-week timepoint, unpaired Student's t-tests. Each dot represents 1 mouse
For (E)–(L) and (P), $n \geq 3$ mice per timepoint (total numbers of clones and mice analyzed per timepoint are given in Table S1).
All images are representative of $n = 3$ mice.

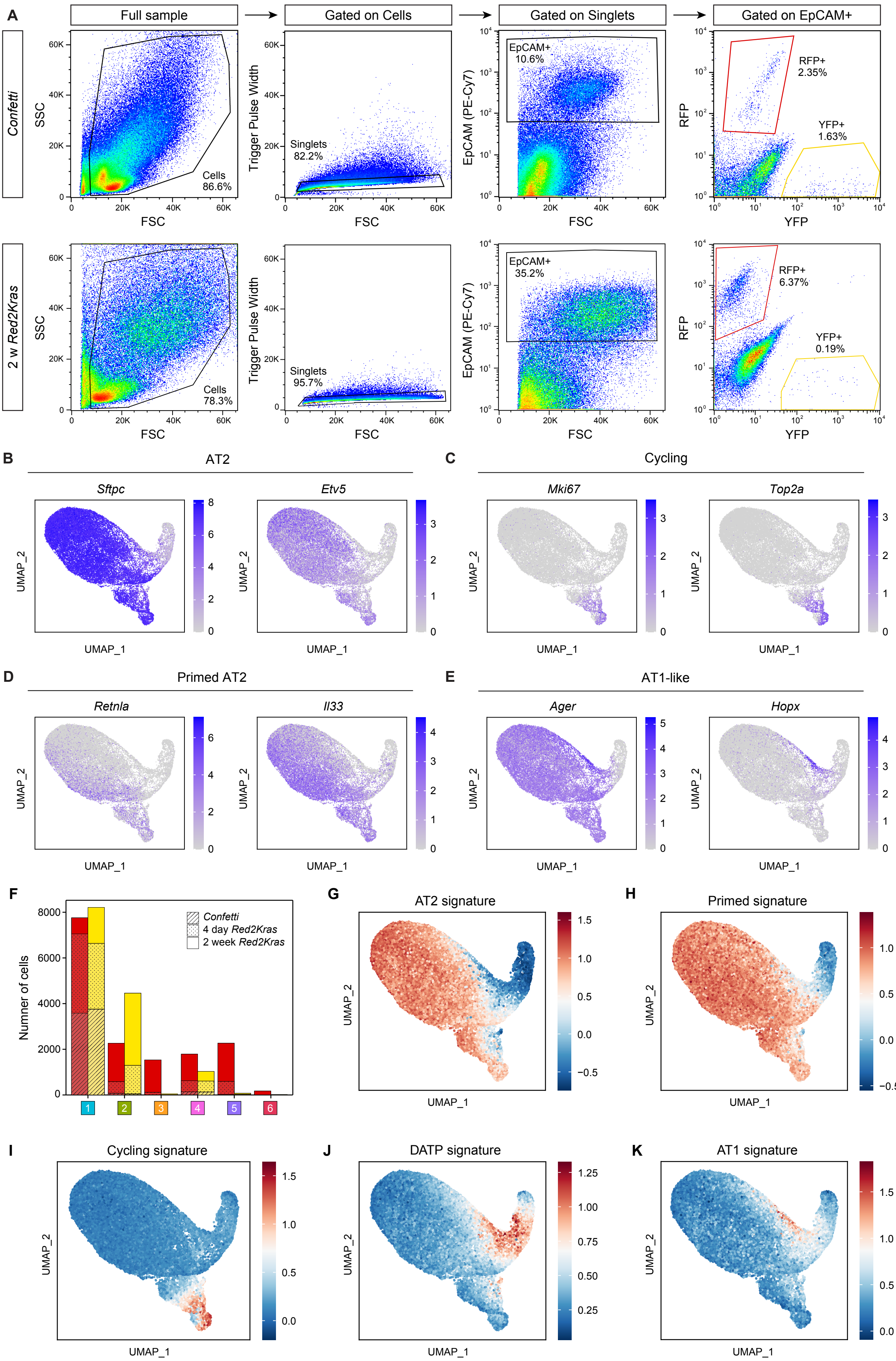

Figure S4. Oncogenic states arising in *Red2Kras* lungs express regenerative state signatures. Related to Figure 3.

(**A**) FACS gating strategy for isolating lineage-labelled (RFP^+ and YFP^+) epithelial cells from *Confetti* (top) and *Red2Kras* (bottom) lungs for scRNA-seq. Samples were first gated on cells and singlets before gating on the $EpCAM^+$ population to sort RFP^+ and YFP^+ cells.
(**B**), (**C**), (**D**), (**E**) UMAP plots showing expression of key genes associated with the AT2 (B), Cycling (C), Primed AT2 (D), and AT1-like (E) states identified in Figure 3B.
(**F**) Proportion of RFP^+ (red bars) and YFP^+ (yellow bars) cells occupying each of the six cell states identified in Figure 3. Bars are shaded according to the timepoint the cells were isolated from (diagonal lines, *Confetti*; dots, 4-day *Red2Kras*; blank, 2-week *Red2Kras*).
(**G**), (**H**), (**I**), (**J**), (**K**), UMAP plots showing the expression of regeneration-associated gene signatures of AT2 (G), Primed (or Activated) (H), Cycling (I), DATP (or PATS or $Krt8^+$ ADI) (J), and AT1 (K) states. Signatures were generated based on the top 100 genes distinguishing each state in regeneration taken from three independent publicly available datasets[1–3] and merged.

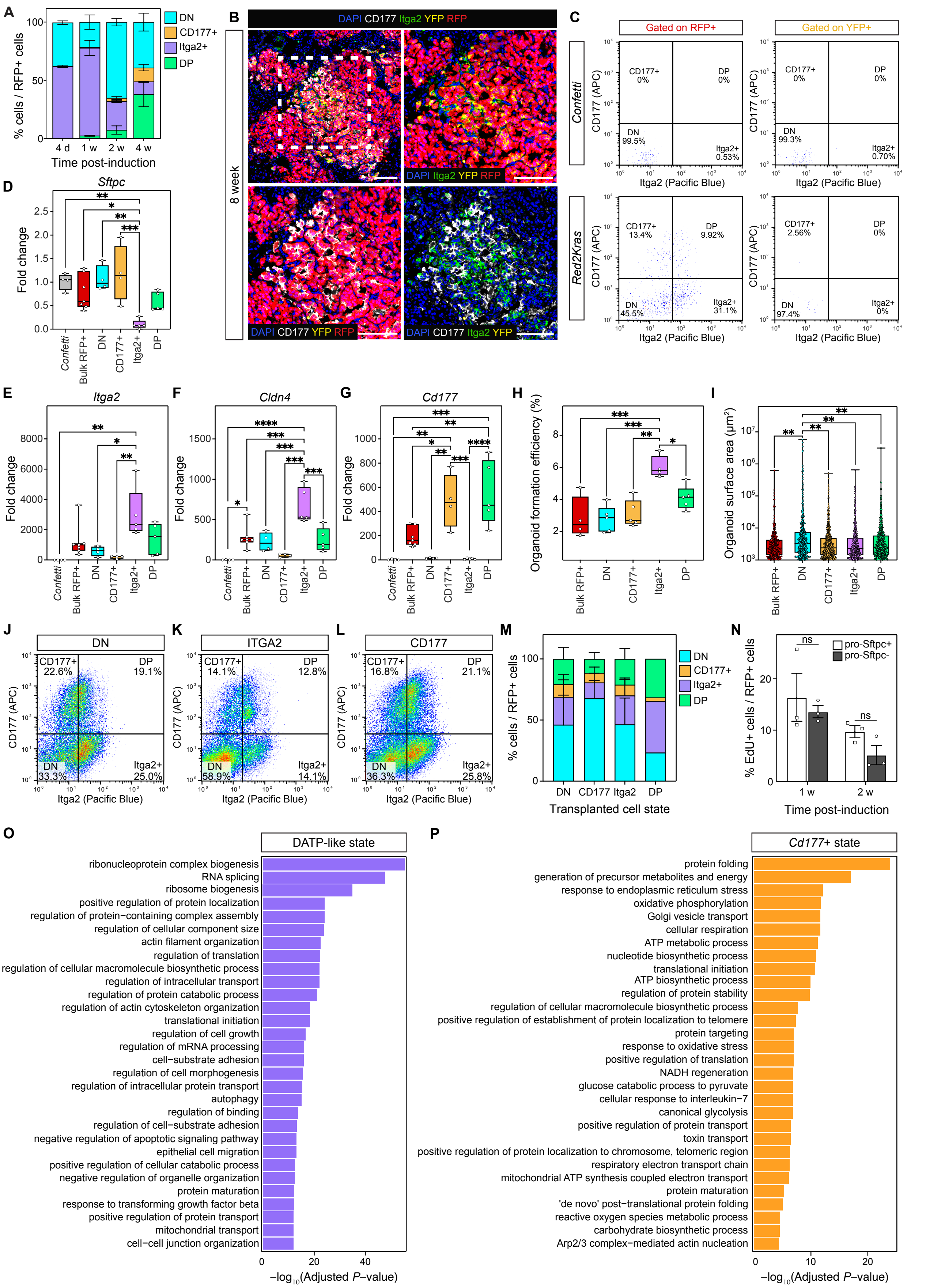

Figure S5. Distinct mutant cellular states persist long-term and undergo reversible transitions resulting in state equipotency. Related to Figure 4.

(**A**) Percentage of RFP^{+} cells occupying each state *in vivo* based on CD177 and Itga2 immunostaining. DN, double negative; DP, double positive for both markers. Data are represented as mean ± SEM. *n* = 2 mice per timepoint.

(**B**) Representative confocal image showing Itga2 (green) and CD177 (white) expression in RFP⁺ cells (top left) in 8-week *Red2Kras* lungs. Magnifications of the highlighted area are given in all other panels. Image representative of Itga2 and CD177 expression in n = 3 mice. DAPI (blue), YFP (yellow), and RFP (red). Scale bars, 100 µm.
(**C**) FACS gating strategy for isolating CD177$^+$ and Itga2$^+$ cells, as well as those double negative (DN) and double positive (DP) for both markers, from the mutant RFP$^+$ population. Samples were gated on cells, singlets, and EpCAM$^+$ cells before gating on RFP$^+$ and YFP$^+$ populations. RFP$^+$ and YFP$^+$ cells in *Confetti* samples were used to establish the DN gate (top), with RFP$^+$ cells in 4-week *Red2Kras* samples localized across all quadrants (bottom).
(**D**), (**E**), (**F**), (**G**) Fold change in mRNA expression of *Sftpc* (D), *Itga2* (E), *Cldn4* (F), and *Cd177* (G) within each mutant state, quantified via qPCR. Values are given as fold change relative to *Confetti* RFP$^+$ control samples, normalized against the housekeeping gene *Oaz1*. Each dot represents 1 independent experiment. Centre line, median; box, interquartile range; whiskers, range. *$p < 0.05$; **$p < 0.01$; ***$p < 0.001$; ****$p < 0.0001$, one-way ANOVA with Tukey's multiple comparisons test.
(**H**) Organoid formation efficiency of independent mutant RFP$^+$ states. Each dot represents 1 independent experiment.
(**I**) 2D surface area of organoids quantified in (H). Each dot represents 1 organoid, with 150 organoids quantified per experiment.
For (H) and (I), data are based on a minimum of n = 4 independent experiments. Centre line, median; box, interquartile range; whiskers, range. *$p < 0.05$; **$p < 0.01$; ***$p < 0.001$, one-way ANOVA with Tukey's multiple comparisons test.
(**J**), (**K**), (**L**) Representative flow cytometric analyses of engrafted mutant cell state identities 2 weeks post-transplantation of DN (J), Itga2$^+$ (K), and CD177$^+$ (L) *Red2Kras* RFP$^+$ cells. Samples were gated on cells, singlets, and live before gating on the EpCAM$^+$ RFP$^+$ population.
(**M**) Quantification of FACS data in (J)–(L) showing the distribution of DN, CD177$^+$, Itga2$^+$, and DP cells within the engrafted RFP$^+$ populations. n = 3 mice transplanted with DN, CD177$^+$, Itga2$^+$ mutant RFP$^+$ cells and n = 1 mouse transplanted with DP mutant RFP$^+$ cells.
(**N**) Percentage of EdU$^+$ cells per mutant clone that were pro-Sftpc$^+$ (white bars) or pro-Sftpc$^-$ (grey bars). Each dot represents 1 mouse. Data are represented as mean ± SEM. ns, not significant; *$p < 0.05$; **$p < 0.01$, paired Student's t-tests.
(**O**), (**P**) Gene ontology (GO) terms significantly enriched in mutant DATP-like (O) and *Cd177*$^+$ mixed (P) gene signatures compared to all other mutant cell states, plotted against their corresponding $-\log_{10}$ adjusted p-values.

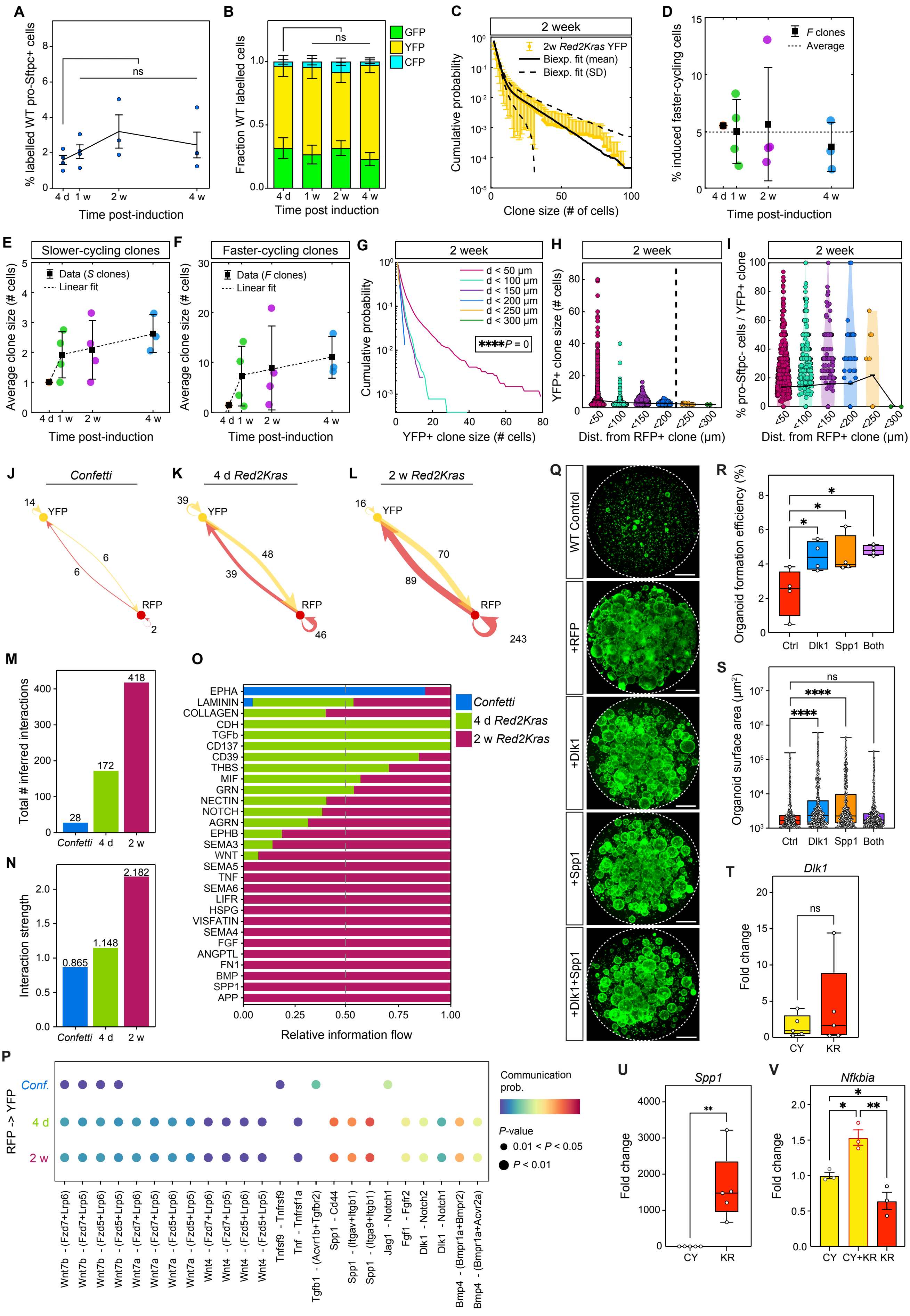

A
% labelled WT pro-Sftpc+ cells
ns
Time post-induction
B
Fraction WT labelled cells
GFP
YFP
CFP
ns
Time post induction
C
2 week
2w Red2Kras YFP
Biexp. fit (mean)
Biexp. fit (SD)
Cumulative probability
Clone size (# of cells)
D
F clones
Average
% induced faster-cycling cells
Time post-induction
E
Slower-cycling clones
Data (S clones)
Linear fit
Average clone size (# cells)
Time post-induction
F
Faster-cycling clones
Data (F clones)
Linear fit
Average clone size (# cells)
Time post-induction
G
2 week
d < 50 μm
d < 100 μm
d < 150 μm
d < 200 μm
d < 250 μm
d < 300 μm
****P = 0
Cumulative probability
YFP+ clone size (# cells)
H
2 week
YFP+ clone size (# cells)
Dist. from RFP+ clone (μm)
I
2 week
% pro-Sftpc- cells / YFP+ clone
Dist. from RFP+ clone (μm)
J
Confetti
K
4 d Red2Kras
L
2 w Red2Kras
YFP
RFP
Q
WT Control
+RFP
+Dlk1
+Spp1
+Dlk1+Spp1
R
Organoid formation efficiency (%)
Ctrl
Dlk1
Spp1
Both
S
Organoid surface area (μm²)
ns
****
****
Ctrl
Dlk1
Spp1
Both
M
Total # inferred interactions
28
172
418
Confetti
4 d
2 w
N
Interaction strength
0.865
1.148
2.182
Confetti
4 d
2 w
O
EPHA
LAMININ
COLLAGEN
CDH
TGFb
CD137
CD39
THBS
MIF
GRN
NECTIN
NOTCH
AGRN
EPHB
SEMA3
WNT
SEMA5
TNF
SEMA6
LIFR
HSPG
VISFATIN
SEMA4
FGF
ANGPTL
FN1
BMP
SPP1
APP
Confetti
4 d Red2Kras
2 w Red2Kras
Relative information flow
T
Dlk1
ns
Fold change
CY
KR
P
RFP -> YFP
Conf.
4 d
2 w
Communication prob.
P-value
0.01 < P < 0.05
P < 0.01
Wnt7b - (Fzd7+Lrp6)
Wnt7b - (Fzd7+Lrp5)
Wnt7b - (Fzd5+Lrp6)
Wnt7b - (Fzd5+Lrp5)
Wnt7a - (Fzd7+Lrp6)
Wnt7a - (Fzd7+Lrp5)
Wnt7a - (Fzd5+Lrp6)
Wnt7a - (Fzd5+Lrp5)
Wnt4 - (Fzd7+Lrp6)
Wnt4 - (Fzd7+Lrp5)
Wnt4 - (Fzd5+Lrp6)
Wnt4 - (Fzd5+Lrp5)
Tnfsf9 - Tnfrsf9
Tnf - Tnfrsf1a
Tgfb1 - (Acvr1b+Tgfbr2)
Spp1 - Cd44
Spp1 - (Itgav+Itgb1)
Spp1 - (Itga9+Itgb1)
Jag1 - Notch1
Fgf1 - Fgfr2
Dlk1 - Notch2
Dlk1 - Notch1
Bmp4 - (Bmpr1a+Bmpr2)
Bmp4 - (Bmpr1a+Acvr2a)
U
Spp1
Fold change
**
CY
KR
V
Nfkbia
Fold change
CY
CY+KR
KR

Figure S6. Mutant RFP⁺ cells cause accelerated proliferation and differentiation in the wildtype AT2 compartment. Related to Figures 5 and 6.

(**A**) Percentage of pro-Sftpc$^+$ wildtype lineage-labeled (GFP$^+$, YFP$^+$, or CFP$^+$) cells relative to all pro-Sftpc$^+$ cells over time. Each dot represents 1 mouse.

(**B**) Proportion of wildtype cells expressing each fluorescent reporter at each timepoint.

For (A) and (B), data are represented as mean ± SEM. ns, not significant relative to the 4-day timepoint, one-way ANOVA with Dunnett's multiple comparisons test.

(**C**) Cumulative size distributions of 2-week *Red2Kras* wildtype YFP$^+$ clones. Black line, biexponential fit; dashed line, SD of fit.

(**D**) Inferred fraction of induced faster-cycling wildtype cells in *Red2Kras* lungs obtained from model fits (see Supplementary Note). Each dot represents 1 mouse. Data are represented as mean ± SD. Dashed line, average value.

(**E**), (**F**), Inferred average sizes of slower-cycling (E) and faster-cycling (F) *Red2Kras* wildtype clones over time. Each dot represents 1 mouse. Data are represented as mean ± SD. Dashed line, linear fits.

(**G**) Cumulative size distributions of 2-week YFP$^+$ clones located at different radii, d, from the nearest mutant clone. Each line represents the average distribution of YFP$^+$ clones located at the given distance in $n = 3$ mice. p value is reported for the d<50 μm radius distribution relative to all others, Kolmogorov-Smirnov test.

(**H**) Sizes of individual 2-week YFP$^+$ clones from distributions in (G). Each dot represents 1 clone. Dashed line indicates the point at which clone sizes become statistically indistinguishable from those in corresponding *Confetti* samples, Kolmogorov-Smirnov tests.

(**I**) Percentage of pro-Sftpc$^-$ cells in 2-week YFP$^+$ clones located within different radii of mutant clones. Each dot represents 1 clone.

For (A)–(I), $n \geq 3$ mice per timepoint (total numbers of clones and mice analyzed per timepoint are given in Table S1).

(**J**), (**K**), (**L**), Number of significant inferred outgoing and incoming cell-cell interactions between RFP and YFP populations in *Confetti* (J), *Red2Kras* 4-day (K), and *Red2Kras* 2-week (L) samples. Weight of each arrow relates to interaction strength, with the total number of significant (p < 0.05) interactions given in the plot.

(**M**), (**N**), Total number (M) and strength (N) of interaction in (J)–(L). Total numbers of each are given above the corresponding bar in the plot.

(**O**), Significantly enriched signaling pathways contributing to the inferred interactions in (J)–(N). Only pathways where RFP cells acted as the signaling source are depicted. Absence of bars indicates no significant enrichment, rather than lack of signaling altogether.

(**P**), Significantly enriched ligand-receptor interactions between RFP and YFP cells in each sample corresponding to the pathways highlighted in (O). Each dot is colored according to the cellular communication probability and sized according to the respective p-value.

(**Q**) Representative fluorescent images of organoids established from *Sftpc*$^+$ (ZsGreen$^+$) wildtype AT2 cells and either cultured alone or cultured in the same well as mutant *Red2Kras* RFP$^+$ cells or treated with Dlk1, Spp1, or both Dlk1 and Spp1. Dotted white circles, edge of the well. Scale bars, 1000 μm.

(**R**) Formation efficiency of organoids in (Q). Each dot represents 1 independent experiment.

(**S**) 2D surface area of organoids in (R). Each dot represents 1 organoid, with 150 organoids quantified per experiment.

For (R) and (S), data are based on $n = 4$ independent experiments. Centre line, median; box, interquartile range; whiskers, range. ns, not significant; *p < 0.05; ****p < 0.0001, one-way ANOVA with Tukey's multiple comparisons test.

(**T**), (**U**) Fold change in mRNA expression levels of *Dlk1* (T) and *Spp1* (U) in wildtype *Confetti* YFP$^+$ (CY) and *Red2Kras* mutant RFP$^+$ (KR) organoids, quantified via qPCR. Values are given as fold change relative to CY control samples normalized against the housekeeping gene *18S*. Each dot represents 1 independent experiment. Centre line, median; box, interquartile range; whiskers, range. ns, not significant; **p < 0.01, paired Student's t-test.

(**V**) Fold change in mRNA expression levels of *Nfkbia* in *Red2Kras* mutant RFP$^+$ organoids (KR) and wildtype *Confetti* YFP$^+$ organoids either cultured alone (CY) or in the same well as *Red2Kras* mutant RFP$^+$ organoids (CY+KR). Values are given as fold change relative to CY controls, normalized against the housekeeping gene *18S*. Each dot represents 1 independent experiment. Data are represented as mean ± SEM. $n = 3$ biological replicates. *p < 0.05; *p < 0.01, unpaired Student's t-tests.

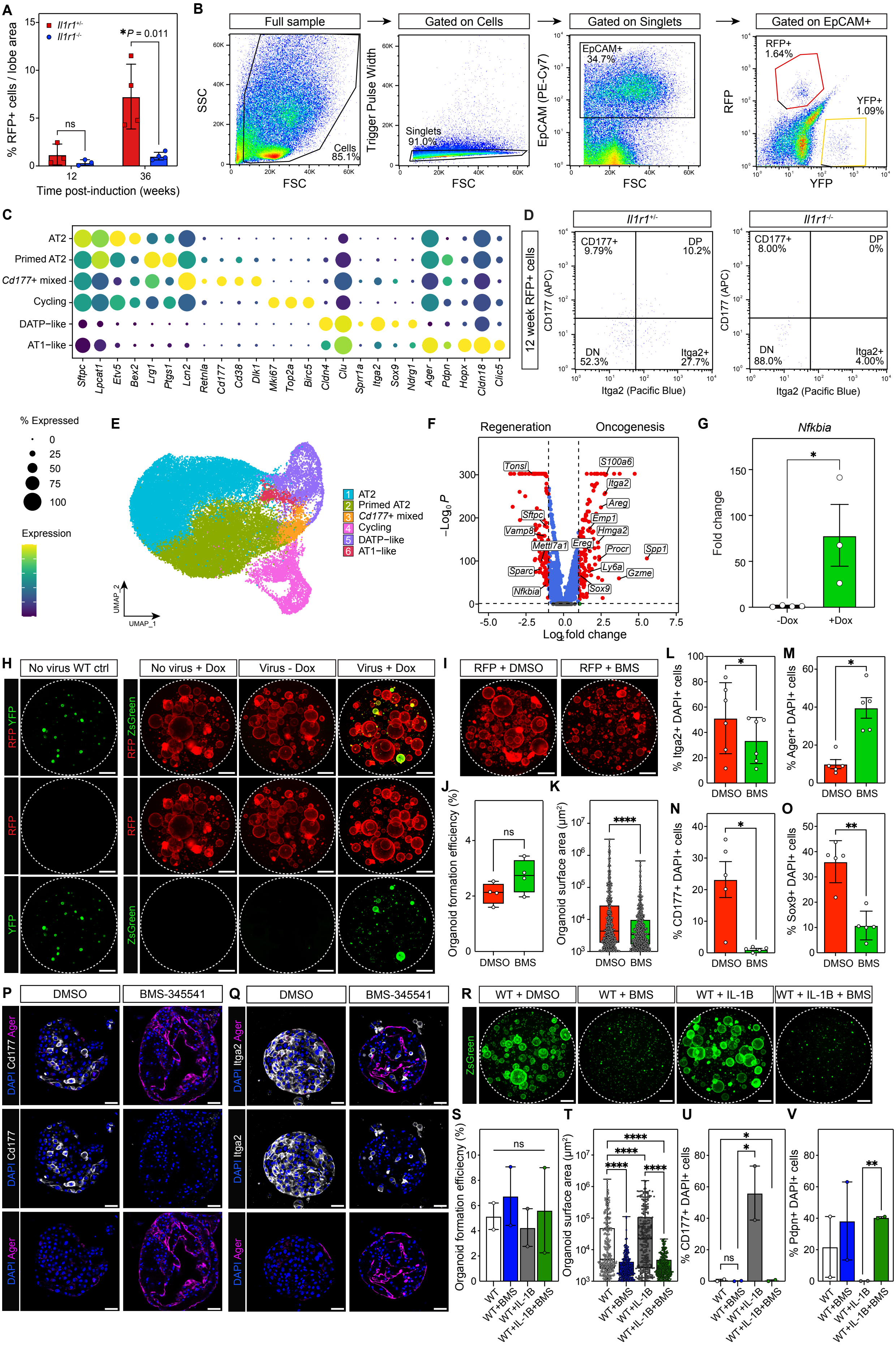

Figure S7. Mutant cells hijack the regeneration program but fail to acquire expression of key NF-κB negative regulators required for AT1 differentiation. Related to Figure 7.

(**A**) Percentage area occupied by mutant RFP$^+$ cells in *Il1r1*$^{+/-}$ *Red2Kras* controls (red bars) versus *Il1r1*$^{-/-}$ *Red2Kras* cohorts (blue bars). Data are represented as mean ± SEM. n = 3 mice per condition. ns, not significant; *p < 0.05, unpaired Student's t-tests.
(**B**) FACS gating strategy for isolating RFP$^+$ and YFP$^+$ cells from *Il1r1*$^{loxP/loxP}$*; Red2Kras* mice. Gating shown for a dissociated 2-week *Il1r1*$^{+/-}$ lung sample. Samples were gated on cells and singlets before gating on the EpCAM$^+$ population to sort RFP$^+$ cells for 10x Genomics sequencing.
(**C**) Expression of key marker genes of distinct states captured in *Il1r1*$^{loxP/loxP}$*; Red2Kras* RFP$^+$ cohorts. Each dot is colored according to expression levels and sized according to the proportion of cells expressing that marker. Similar transcriptomic signatures to those in Figure 3 were seen.
(**D**) Flow cytometric analyses of CD177 and Itga2 expression in 12-week *Il1r1*$^{+/-}$ control (left) and *Il1r1*$^{-/-}$ (right) mutant RFP$^+$ cells. Samples were gated on cells, singlets, and EpCAM$^+$ epithelial cells before the gates illustrated here.
(**E**) UMAP plot showing all integrated 40,804 single-cell transcriptomes collected from our previously published regeneration dataset[1] and our oncogenic dataset in Figure 3, clustered into the six identified cellular states. Cells are colored and numbered by state.
(**F**) Volcano plot showing differentially expressed genes between regeneration-associated DATPs (left, n = 811 cells) and oncogenesis-associated DATP-like cells (right, n = 2347 cells). Genes in red were differentially expressed to a significant level (Wilcoxon-signed rank test), with genes of interest specifically highlighted.
(**G**) Fold change in mRNA expression levels of *Nfkbia* after lentiviral transduction with the *Nfkbia* overexpression vector construct. Values are given as fold change relative to virally transduced mutant organoids without doxycycline treatment, normalized against the housekeeping gene *Oaz1*. Each dot represents 1 independent experiment. Data are represented as mean ± SEM. n = 4 control samples (-Dox) and n = 3 ZsGreen$^+$ samples (+Dox). *p < 0.05, unpaired Student's t-test.
(**H**) Representative fluorescent images of organoids established from *Red2Kras* YFP$^+$ (left) and RFP$^+$ (right) cells transduced with lentiviral vectors where indicated and cultured either with or without doxycycline. Dotted white circles, edge of the Matrigel dome. Scale bars, 1000 µm.
(**I**) Representative fluorescent images of organoids established from mutant RFP$^+$ cells and treated either with DMSO or BMS-345541. Dotted white circles, edge of the Matrigel dome. Scale bars, 1000 µm.
(**J**) Formation efficiency of DMSO-treated (red) and BMS-treated (green) mutant RFP$^+$ organoids. Each dot represents 1 independent experiment.
(**K**) 2D surface area of organoids in (J). Each dot represents 1 organoid, with 150 organoids quantified per experiment.
For (J) and (K), data represent n = 4 independent experiments. Centre line, median; box, interquartile range; whiskers, range. ns, not significant; ****p < 0.0001, paired Student's t-tests.
(**L**), (**M**), (**N**), (**O**) Percentage of Itga2$^+$ (L), Ager$^+$ (M), CD177$^+$ (N), and Sox9$^+$ (O) cells in mutant RFP$^+$ organoids treated with either DMSO (red) or BMS (green) for 7 days *in vitro*. Data are represented as mean ± SEM. *p < 0.05; **p < 0.01, paired Student's t-tests.
(**P**), (**Q**) Representative confocal images of wholemount RFP$^+$ organoids in (I) showing acquisition of AT1 marker Ager (magenta) with a corresponding loss of CD177 (white, (P)) and Itga2 (white, (Q)) following BMS treatment. DAPI (blue). Scale bars, 50 µm.
(**R**) Representative fluorescent images of organoids established from wildtype ZsGreen$^+$ AT2 cells and treated either with DMSO, BMS-345541, IL-1β, or both BMS-345541 and IL-1β. Dotted white circles, edge of the Matrigel dome. Scale bars, 1000 µm.
(**S**) Formation efficiency of organoids in (R). Each dot represents 1 independent experiment. Data are represented as mean ± SEM.
(**T**) 2D surface area of organoids in (S) Each dot represents 1 organoid, with 150 organoids quantified per experiment. Centre line, median; box, interquartile range; whiskers, range.
For (S) and (T), data represent n = 2 independent experiments. ns, not significant; *p < 0.05; *p < 0.01; ****p < 0.0001 relative to other conditions as shown, paired Student's t-tests.
(**U**), (**V**) Percentage of CD177$^+$ (U) and Pdpn$^+$ (V) cells in wildtype ZsGreen$^+$ organoids in (R). Data are represented as mean ± SEM. *p < 0.05; **p < 0.01, paired Student's t-tests.

Table S1. Total numbers of clones and mice analyzed per timepoint. Related to Figures 1, 2, 5, and 6.

Sftpc-CreERT2; R26R-Confetti samples								
Timepoint	Reporter	Number of clones captured and analyzed (excluding singlets)						
		Mouse 1	Mouse 2	Mouse 3	Mouse 4	Mouse 5	Total	Combined
1 w	YFP	430	197	603	-	-	1230	3460
	RFP	986	323	921	-	-	2230	
2 w	YFP	56	51	381	-	-	488	1649
	RFP	272	153	736	-	-	1161	
4 w	YFP	473	253	340	233	138	1437	3887
	RFP	729	329	659	403	330	2450	
12 w	YFP	657	569	305	-	-	1531	4439
	RFP	1158	1200	550	-	-	2908	
24 w	YFP	199	966	72	-	-	1237	3553
	RFP	511	1595	210	-	-	2316	
36 w	YFP	144	111	296	-	-	551	2995
	RFP	847	100	1497	-	-	2444	
52 w	YFP	159	266	100	-	-	525	1556
	RFP	329	368	334	-	-	1031	
60 w	YFP	461	117	342	-	-	920	2626
	RFP	724	300	682	-	-	1706	
72 w	YFP	270	136	92	174	-	672	1916
	RFP	470	258	148	368	-	1244	

Sftpc-CreERT2; Red2Kras samples							
Timepoint	Reporter	Number of clones captured and analyzed (excluding singlets)					
		Mouse 1	Mouse 2	Mouse 3	Mouse 4	Mouse 5	Total
4 d	YFP (wildtype)	892	7	38	-	-	937
1 w		4532	1586	4848	-	-	12073
2 w		4155	342	1142	-	-	10464
4 w		3107	1811	2051	-	-	6969
4 d	RFP (mutant)	886	11	25	-	-	10464
1 w		2967	138	4800	-	-	8756
2 w		1253	252	613	-	-	4857
4 w		1086	544	870	-	-	2500

Table S2. List of antibodies used and their respective dilutions. Related to STAR Methods.

Antibody name	Supplier	Dilution
**PRIMARY ANTIBODIES**		
Rat anti-Ager	R&D Systems	1:200
Rabbit anti-caveolin-1 (D46G3)	Cell Signaling Technology	1:100
Rabbit anti-CD177	R&D Systems	1:300
Rabbit anti-cleaved caspase-3 (Asp175)	Cell Signaling Technology	1:100
Rat anti-CD49b (Itga2) (DX5)	Thermo Fisher Scientific	1:100
Rabbit anti-Itga2 (EPR17338)	Abcam	1:200
Rat anti-Ki67 (SolA15)	Thermo Fisher Scientific	1:100
Rat anti-cytokeratin 8 (Krt8)	DSHB	1:300
Rat anti-DC-LAMP (Lamp3 / CD208)	Dendritics	1:100
Rabbit anti-Lpcat1	Proteintech	1:100
Hamster anti-T1a (Pdpn)	DSHB	1:300
Rabbit anti-phospho-ERK	Cell Signaling Technology	1:100
Rabbit anti-prosurfactant protein C	Millipore	1:300
Rabbit anti-RasG12D mutant (D8H7)	Cell Signaling Technology	1:100
Goat anti-CC10 (Scgb1a1) (T-18)	Santa Cruz Biotechnology	1:200
Goat anti-SPC (Sftpc)	Santa Cruz Biotechnology	1:100
Rabbit anti-Sox9 (EPR14335)	Abcam	1:300
Goat anti-tdTomato	SICGEN	1:200
**SECONDARY ANTIBODIES**		
Alexa Fluor® 647 donkey anti-Goat IgG (H+L)	Thermo Fisher Scientific	1:500
Alexa Fluor® 647 goat anti-Syrian Hamster IgG (H+L)	Thermo Fisher Scientific	1:500
Alexa Fluor® 647 donkey anti-Rabbit IgG (H+L)	Thermo Fisher Scientific	1:500
Alexa Fluor® 488 donkey anti-Rat IgG (H+L)	Thermo Fisher Scientific	1:500
DyLight 755 donkey anti-Goat IgG (H+L)	Thermo Fisher Scientific	1:200
DyLight 755 donkey anti-Rabbit IgG (H+L)	Thermo Fisher Scientific	1:200
DyLight 755 donkey anti-Rat IgG (H+L)	Thermo Fisher Scientific	1:200
**FLOW CYTOMETRY ANTIBODIES**		
PE/Cyanine7 anti-mouse CD326 (Ep-CAM)	BioLegend	1:200
Alexa Fluor® 647 rat anti-mouse CD177	BD Biosciences	1:200
Pacific Blue™ anti-mouse CD49b (Itga2)	Biolegend	1:200
APC Rat Anti-Mouse CD45	BD Biosciences	1:200
APC Rat Anti-Mouse CD31 (MEC13.3)	BD Biosciences	1:200
FITC MHC Class II (I-A/I-E) monoclonal antibody (M5/114.15.2)	eBioscience	1:200

Table S3. List of mouse primers used for qPCR. Related to STAR Methods.

ID	Sequence
Oaz1 Forward	GCCAATGAACGAGATCACTT
Oaz1 Reverse	GCTGTTTAAGATGGTCAGGTGA
Sftpc Forward	CAAACGCCTTCTCATCGTGGTTGT
Sftpc Reverse	TTTCTGAGTTTCCGGTGCTCCGAT
Itga2 Forward	TACAGACGTGCTCCTGGTAGGT
Itga2 Reverse	CCGAGCATTTCCAGTGCCTTCT
Cldn4 Forward	GTCCTGGGAATCTCCTTGGC
Cldn4 Reverse	TCTGTGCCGTGACGATGTTG
Cd177 Forward	GCAATGACCTGTCTACCACAGC
Cd177 Reverse	CGGTGCATTCTCACAGGCTTGT
18S Forward	CGGCTACCACATCCAAGGAA
18S Reverse	GCTGGAATTACCGCGGCT
Sox9 Forward	AGGAAGCTGGCAGACCAGTA
Sox9 Reverse	TCCACGAAGGGTCTCTTCTC
Spp1 Forward	GCTTGGCTTATGGACTGAGGTC
Spp1 Reverse	CCTTAGACTCACCGCTCTTCATG
Dlk1 Forward	TGGCTGTGTCAATGGAGTCTGC
Dlk1 Reverse	CCACGCAAGTTCCATTGTTGGC

SUPPLEMENTAL INFORMATION REFERENCES

1. Choi, J., Park, J.-E., Tsagkogeorga, G., Yanagita, M., Koo, B.-K., Han, N., and Lee, J.-H. (2020). Inflammatory Signals Induce AT2 Cell-Derived Damage-Associated Transient Progenitors that Mediate Alveolar Regeneration. Cell Stem Cell *27*, 366-382.e7. https://doi.org/10.1016/j.stem.2020.06.020.
2. Kobayashi, Y., Tata, A., Konkimalla, A., Katsura, H., Lee, R.F., Ou, J., Banovich, N.E., Kropski, J.A., and Tata, P.R. (2020). Persistence of a regeneration-associated, transitional alveolar epithelial cell state in pulmonary fibrosis. Nat Cell Biol *22*, 934–946. https://doi.org/10.1038/s41556-020-0542-8.
3. Strunz, M., Simon, L.M., Ansari, M., Kathiriya, J.J., Angelidis, I., Mayr, C.H., Tsidiridis, G., Lange, M., Mattner, L.F., Yee, M., et al. (2020). Alveolar regeneration through a Krt8+ transitional stem cell state that persists in human lung fibrosis. Nat Commun *11*, 3559. https://doi.org/10.1038/s41467-020-17358-3.
